# A Systematic Review to Inform the Development of a Reporting Guideline for Concept Mapping Research

**DOI:** 10.3390/mps6050101

**Published:** 2023-10-17

**Authors:** Sandesh Pantha, Martin Jones, Pragya Gartoulla, Richard Gray

**Affiliations:** 1 School of Nursing and Midwifery, La Trobe University, Bundoora, VIC 3086, Australia; r.gray@latrobe.edu.au; 2 Department of Rural Health, University of South Australia, Whyalla Campus, Whyalla Norrie, SA 5608, Australia; martin.jones@unisa.edu.au; 3 Australian Institute of Family Studies, Melbourne, VIC 3000, Australia; pragya.gartoulla@aifs.gov.au

**Keywords:** concept mapping, quality, reporting guidelines, systematic review

## Abstract

Concept mapping is a phased, mixed-method approach that is increasingly used in health research to develop an understanding of complex phenomena. The six phases of concept mapping are preparation, idea generation, structuring (clustering and prioritization), data analysis, interpretation, and utilization of the map. The reporting of concept mapping research requires the development of a specific reporting guideline. We conducted a systematic review to identify candidate reporting items for inclusion in a reporting guideline. Three databases (MEDLINE, CINAHL, and PsycInfo) were searched to identify studies that used concept mapping methodology. We included 75 concept mapping studies published since 2019 from which we extracted information about the quality of reporting. A third of the studies focused on public health. We identified 71 candidate items that relate to the quality of reporting concept mapping research. The rationale for the study, the focus prompt, procedures for brainstorming, and structuring statements were consistently reported across the included studies. The process for developing the focus prompt, the rationale for the size of the stakeholder groups, and the process for determining the final concept map were generally not reported. The findings from the review will be used to inform the development of our reporting guideline for concept mapping research.

## 1. Introduction

Concept mapping is a specific type of mixed-method research that involves qualitative and quantitative procedures for data collection and analysis [1,2]. Concept mapping is used to develop deep a understanding of a complex phenomenon in a visual form [3]. Our manuscript focuses on the concept mapping methodology described by Trochim [1,4,5,6]. There are other approaches to generate concept maps, such as Novak’s method [7]; however, this is not the focus of this manuscript. There are six phases in concept mapping—preparation, idea generation, structuring of statements, data analysis, interpretation, and utilization of the map [1].

The quality of reporting concept mapping studies has not been evaluated comprehensively. Two systematic reviews have examined—albeit in a limited way—the quality of reporting of concept mapping research [8,9]. For example, Rosas and Kane [8] reported a review of 69 concept mapping studies using the Concept Systems (www.groupwisdom.com, accessed on 10 April 2022) software package and reported that authors frequently omitted important information about the research, including the flow of participants through the study and stress value, a measure of the fit between the final concept map and how participants sorted the data [8]. Donnelly [9] conducted a systematic review of 104 doctoral dissertations that used concept mapping, reporting that the included studies did not provide a comprehensive description of the methods that they applied in their research. The authors of both reviews argue a need to improve the quality of reporting of concept mapping research and suggest that a reporting guideline may be helpful in achieving this [8,9].

Guidelines are intended to enhance the quality and transparency of research reporting [10]. Generally, taking the form of a list of recommendations, reporting guidelines ensure that authors provide a detailed description of the design, conduct, and reporting of research [11] so that consumers of research (researchers, clinicians, the public) can appraise the quality of the work and determine its relevance to health care practice [12]. The first reporting guideline, CONsolidated Standards Of Reporting Trials (CONSORT), was described in 1996 [13]. Subsequently, there has been a rapid expansion in the number of reporting guidelines across a range of different research methodologies, including observational (STROBE [14], TRIPOD [15], STARD [16]), qualitative (COREQ [17], SRQR [18]), and mixed-method (GRAMMS [19]) designs.

Several authors have investigated compliance with reporting guidelines in published manuscripts [20,21,22,23,24]. For example, Ziemann et al. (2022) evaluated adherence to the STROBE checklist (Strengthening The Reporting of OBservational studies in Epidemiology) in observational studies of COVID-19 treatments [21]. The review authors reported that around half of the checklist items were addressed in the included studies [21]. Participant recruitment, potential sources of bias, and study limitations were not reported in about 9 out of 10 studies [21]. Walsh et al. [22] examined compliance with the 32-item COREQ (COmprehensive REporting of Qualitative studies) checklist in a review of 197 qualitative studies published in nursing journals [22]. Adherence with the reporting guidelines was rated depending on the number of items addressed in the manuscript (good ≥25, moderate 17–24, poor 9–16, and very poor ≤8). Compliance with the reporting guidelines was rated moderate in half of the included studies [22].

The central repository for reporting guidelines in health research is the EQUATOR (Enhancing the QUAlity and Transparency Of health Research) network. The EQUATOR is a searchable repository of 550 reporting guidelines (equator-network.org, accessed on 28 November 2022) across different methodologies. We searched the EQUATOR network for mixed-method and concept mapping reporting guidelines. None of the 16 reporting guidelines related to mixed-method research (www.equator-network.org, accessed on 28 November 2022) had a specific focus on concept mapping design.

The “concept systems” software package website (www.groupwisdom.com/gcmrg assessed on 10 April 2022) has a 10-point checklist to inform reports of concept mapping studies. The checklist is limited to studies using the concept systems software package. In addition, the process of developing the checklist has not been reported.

Given the complexity and specific methodological procedures for concept mapping, there is a robust scientific justification for developing a precise reporting guideline. Moher et al. [25] describe the process for developing a reporting guideline for inclusion in the EQUATOR network. A review of the existing literature to identify the need for a reporting guideline, determine the quality of reporting, and generate a list of potential candidate items is a pre-requisite for the development of a reporting guideline [25].

### Aim of the Review

Our systematic review has two aims:

1. Review the quality of reporting of concept mapping studies to establish key areas where reporting may be sub-optimal.
2. Identify candidate items for inclusion in the reporting guideline for concept mapping research.

## 2. Method

The reporting of our review adheres to the Preferred Reporting guidelines for Systematic review and Meta-Analysis (PRISMA) [26] (Supplementary Document S1 is a copy of the completed PRISMA checklist). We prospectively registered our protocol for developing a concept mapping reporting guideline with the Open Science Framework (OSF) (https://osf.io/h54k6/, accessed on 1 July 2023) on 17 July 2021. Our study is listed with the EQUATOR network as a guideline under development (https://www.equator-network.org, accessed on 1 July 2023). Additionally, we published a study protocol for developing concept mapping reporting guidelines, including the details of the systematic review methodology we intended to follow [27].

### 2.1. Eligibility Criteria

We included any study where the authors indicated that they followed the concept mapping methodology described by Trochim (1989) [1] and where the focus of the study was on a health topic. Studies were included in the review if they were published in English in a peer-reviewed journal. No date restrictions were applied.

### 2.2. Information Sources

Three online databases—Medical Literature Analysis and Retrieval System (MEDLINE), PsycInfo, and Cumulative Index to Nursing and Allied Health Literature (CINAHL)—were searched through the Ovid platform. Although this is the minimum number of databases recommended by Cochrane when conducting a systematic review, we considered that to achieve the aims of our review, these databases would capture most of the relevant concept mapping research. Our initial search was carried out on 8 June 2021. We contemplated updating our initial search; however, the Cochrane handbook suggests (p. 89) that if a substantial number of papers are generated from the initial search, there is little value to conducting additional further searches [28].

### 2.3. Search Strategy

Donnelly [9] reported a systematic review of the quality of reporting of concept mapping in doctoral dissertations. The search concepts that we used were derived from this work. The search constructs ‘concept map*’, ‘structured conceptualization’, ‘concept systems’, and ‘Aridane’ were combined with the bullion operator ‘OR’. Our search was initially developed for MEDLINE and subsequently tailored for other databases. Complete search strategies for the three databases can be accessed as a supplementary Document (Supplementary Document S2).

### 2.4. Selection Process

We used Covidence, an online software package for systematic reviews, to support our research. Citations from the three databases were exported to Covidence, and duplicate citations removed. Two reviewers (SP, PG) completed title and abstract and full-text screening, again in Covidence, against eligibility criteria. A third reviewer resolved conflicts (RG). If there were multiple papers from the same study, as per the Cochrane handbook we included the first reported paper [28].

### 2.5. Data Collection Process

Our data extraction tool was developed based on the detailed description of concept mapping provided by Trochim [1,4,5,6]. One researcher (SP) completed data extraction against our 46-item data extraction spreadsheet [27]. All data items were coded ‘Reported’, ‘Not Reported’, or ‘Not applicable’. After completing data extraction for the first ten studies, we noted that important information about the reporting of concept mapping was not being captured. Consequently, we revised our data extraction form, adding 44 additional items, for example, whether authors indicated that they used a reporting guideline when drafting the manuscript, stakeholder involvement in statement reduction, and data interpretation. The final data extraction form has 90 items, of which 19 relate to study characteristics and 71 to the quality of reporting. Both data extraction forms are available as supplementary Documents (Supplementary Documents S3 and S4).

### 2.6. Synthesis Method

Characteristics of included studies were summarized using standard descriptive statistics. We reported the number and percentage of manuscripts that addressed each data item. Some data items did not apply to all included studies (for example, some studies did not include a focus prompt because statements were generated from a secondary source [a literature review]); this reduced the number of studies in the denominator. The number of studies used as the denominator is noted in the data extraction table.

## 3. Results

### 3.1. Study Selection

**Figure 1:**
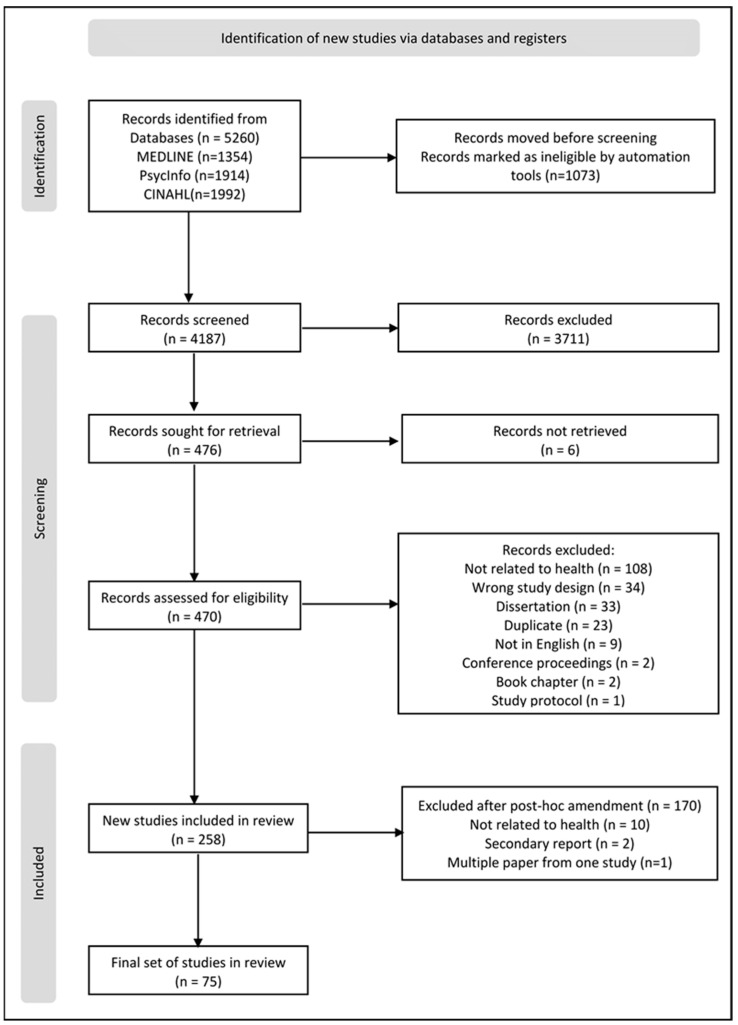
PRISMA flowchart.

Figure 1 (PRISMA flowchart) shows the flow of studies through the different phases of our review. Our search identified 5260 citations, of which 258 met the inclusion criteria. Supplementary Document S5 is a list of all 258 included studies.

### 3.2. Amendments to the Review Protocol

We had 258 studies for data extraction. Given that this review aimed to examine the quality of reporting of concept mapping research and generate candidate items for inclusion in a reporting guideline, we considered that it was not necessary or feasible to extract data from all 258 papers to address the aim of the review. Therefore, we made a post hoc amendment to the inclusion criteria in our protocol to extract data only from studies published after 2018 (2019–2021, three years). This was primarily performed to limit the number of published concept mapping studies for data extraction. Our revised inclusion criteria limited the number of papers included in the review to 88 (Supplementary Document S6).

### 3.3. Studies Excluded during Data Extraction

During data extraction, we excluded an additional thirteen papers (Supplementary Document S7). Ten did not have a health focus, and two were secondary reports. Two manuscripts reported results from a single study (possible duplicate publication) [29,30]; we included the first paper (by date of submission) [29]. As the overlap between these two papers was substantial, we informed the editors of both journals on 4 January 2023. As of 30 June 2023, we had not received a response. The final list of 75 included studies can be accessed in Supplementary Document S8.

### 3.4. Characteristics of Included Studies

Table 1 shows the characteristics of the 75 included studies. Between one and five stakeholder groups were involved in the included studies (mean = 1.7, *SD* = 0.9). Published studies had a focus predominantly in public health, occupational health, and rehabilitation. Two-thirds of the included studies were conducted in Europe or North America.

The authors of 12 studies did not report the total number of participants in the study. The average number of statements used for structuring (prioritization and clustering) was 94. The final concept map, on average, had seven clusters. Seven studies included reports of the peer-review process [31,32,33,34,35,36,37]. The authors of one study indicated that they followed a reporting guideline when preparing their manuscript [38]. Two studies were prospectively registered [38,39] and an equal number reported publishing a study protocol [38,40].

### 3.5. Quality of Reporting of Concept Mapping Studies

There were seventy-one data extraction items related to the reporting of concept mapping research. We presented data items under eight subheadings: 1. Title and abstract, 2. Background, 3. Methods, 4. Ethics, 5. Results, 6. Discussion, 7. Limitations, and 8. Registration and study protocol. Table 2 is a summary of the quality of reporting of the included studies. The full dataset is available as Supplementary Document S4.

#### 3.5.1. Title and Abstract (12 Items)

The authors of two-thirds of the included studies stated, in the title of the manuscript, that they were reporting concept mapping research. Virtually all studies provided a scientific justification for the study and reported details of the stakeholder groups involved in the abstract. One in four studies gave information on the focus prompt and details of where fieldwork for the study was conducted.

#### 3.5.2. Background (Three Items)

Most studies reported the aim of the study. A rationale for concept mapping as a study design was included in half of the papers.

#### 3.5.3. Methodology

Twenty-five data items were related to the reporting of concept mapping methods, organized under four subheadings (which related to the first four phases of concept mapping): preparation, idea generation, structuring (clustering and prioritization), and data-analysis.

##### Preparation (Eight Items)

Essentially all study authors reported a focus prompt for their research and gave a description of the study stakeholder groups. Fewer than a quarter of studies gave a rationale for selecting stakeholder groups for the research. The authors of 19 studies reported the processes they followed to develop the focus prompt; of these, 15 (79%) reported how stakeholders were engaged in the work.

##### Idea Generation (Five Items)

A description of the idea generation (brainstorming and statement reduction) procedures was included in most of the included studies. A rationale for the sample size was reported in a third of the studies. Three-quarters of studies reported detailed processes for idea synthesis.

##### Structuring of Statements (Six Items)

Details about whether the structuring of statements was conducted online, face-to-face, or hybrid was reported in almost all included studies. A rationale for the number of participants required for this phase of concept mapping was reported in around a third of studies.

##### Data Analysis (Six Items)

The three steps in the analysis of concept mapping data (binomial distance matrix, multidimensional scaling, and cluster analysis) were generally described by all study authors. Stakeholder engagement in data analysis was reported in half of the studies. The authors of one in three studies did not outline the process for determining the final cluster solution.

#### 3.5.4. Additional Information (Four Items)

Information on the Institutional Review Board (IRB) or ethics committee that provided approval for the research was reported in more than eight out of ten studies. The ethics approval number was reported in half of the studies. Three-quarters of the studies stated the process of obtaining consent from the study participants.

#### 3.5.5. Results

There were twenty-two data items related to the reporting of results of concept mapping studies, and these were detailed under three subheadings: participants, statements, and clusters.

##### Participants (Seven Items)

The demographic and clinical (where relevant) characteristics of participants were described in 68 studies. Nine out of ten studies reported the number of people who participated in idea generation (brainstorming) and the structuring of statements (prioritization and clustering). The participant response rate (the percentage of invited people who participated) was reported in half of the included studies.

##### Statements Generated (Six Items)

Three-quarters of the studies reported the total number of statements generated by participants during the idea generation (brainstorming) phase of the research. A complete list of included statements was reported in 90% of studies. Less than a third (n = 20, 29%) of the 68 studies (that had a rating task) reported scores for each of the included statements.

##### Clusters (Nine Items)

Cluster labels were reported in all studies. The authors of fewer than a quarter of the included studies reported information on the average number of clusters generated by participants.

#### 3.5.6. Discussion (Three Items)

The authors of all studies appropriately considered the findings of their work within broader scientific literature. Half of the studies reported how the findings of the research might be utilized in clinical practice. A summary of the key findings from the study was reported in eight out of ten studies.

#### 3.5.7. Limitations (One Item)

The limitations of the study were fully described and discussed by most authors of the included concept mapping studies.

#### 3.5.8. Registration and Study Protocol (One Item)

The authors of three studies reported that they had registered their study or had published the study protocol.

## 4. Discussion

The aim of our systematic review was to describe the quality of reporting concept mapping research and identify potential candidate items to be included in a reporting checklist. Data were extracted from 75 studies published between 2019 and 2021. Overall, the authors of concept mapping research omitted important information about the conduct and reporting of different phases of concept mapping. Specifically, it was common for studies to omit a rationale for including stakeholder groups, details of participant inclusion/exclusion criteria, and information on how stakeholders were engaged in the development of the focus prompt and reduction of statements (idea synthesis). Our findings are consistent with the two previous reviews evaluating the quality of the reporting of concept mapping studies [8,9]. However, these two reviews had a discrete focus. Rosas and Kane (2012) examined studies conducted using a specific concept mapping software package [8]. Donnelly (2017) exclusively reviewed doctoral dissertations using concept mapping methodology [9]. Our study is the first review that looked at health research broadly, extending the evidence on the quality of reporting concept mapping research. The review identified potential areas for improvement in the reporting of concept mapping. Enhancing the quality of reporting enables readers to critically appraise the work [41]. The findings of this and previous reviews potentially provide a strong rationale for developing a guideline to enhance the quality and completeness of reporting concept mapping research in health.

More than half of the studies included in the data extraction were carried out using Concept Systems software. However, none of the studies that used this software followed the Concept Systems’ reporting checklist. One of the reasons for non-compliance with the checklist could be that it is not listed on the EQUATOR network, which is the publicly available repository of the reporting guidelines.

One of the aims of the review was to identify candidate items to include in a reporting guideline for concept mapping research. Moher et al. [25] suggest that a comprehensive literature review is an important part of the guideline development process, informing the identification of candidate items for inclusion in the final document. It has been common when describing the development of reporting guidelines for authors to provide scant details of the literature review that underpinned guideline development (see, for example, [15,17,42,43,44]). Tong et al. [17] report that they conducted a literature review to inform the development of the COREQ (COmprehensive REporting of Qualitative studies) reporting guidelines but did not provide a detailed explanation of how the findings from the review were incorporated in the development of the checklist [17]. Our review is intended to be consistent with the Moher et al. [25] recommendation for guideline development, providing a comprehensive and transparent summary of the strengths and limitations of the reporting of concept mapping research and candidate items for inclusion.

Our manuscript focuses on the quality of the reporting of concept mapping described by Trochim [1,4,5,6]. We acknowledge that there are other techniques to generate concept maps, such as relational concept maps, as described by Novak [7]. Novak and other colleagues have acknowledged practical differences in various approaches of concept mapping [3]. We want to reinforce that our study does not capture information on the quality of the reporting of other approaches to concept maps.

## 5. Limitations of the Study

Our review has some important limitations that warrant consideration. We made two major post hoc amendments to our study protocol that may have impacted the findings of our review. First, we amended our review inclusion criteria to only include studies published from 2019. This was performed primarily to restrict the number of studies in the review, as we identified substantially more studies than predicted. We would expect more complete reporting of studies published more recently, in part because of the movement to encourage the use of reporting guidelines [41], and, consequently, our observations about the quality of the reporting of concept mapping research may be an overestimate. Second, we made substantial amendments to our data extraction template whilst reviewing the included papers, potentially introducing bias into how we determined which data items to include. It would have been preferential if we had undertaken a pilot data extraction exercise to comprehensively determine the data items we intended to use for this review.

Data extraction was performed by one researcher. It would have been more rigorous if two researchers had completed this task and checked the consistency of ratings. We acknowledge this is a limitation of our review methodology. We did not update our search. As per Cochrane handbook recommendations, it would have been ideal to update the search after 12 months of the initial search. However, given that the aim of the study was to evaluate the quality of research reporting, updating the search, we argue, would have provided little additional value to the review.

## 6. Conclusions

Our systematic review evaluated the quality of reporting concept mapping studies in health research and identified candidate items for inclusion in a reporting guideline. Our systematic review evaluated 258 studies, of which we extracted data from 75 manuscripts published after 2018. The 71 items that relate to the quality of reporting will be used to inform the development of a reporting guideline for concept mapping research.

## Figures and Tables

**Table 1:** Characteristics of included studies (n = 75 unless otherwise stated).

Characteristic	Mean (*SD*)	Range	Number (%)
Stakeholder groups	1.7 (0.9)	1–5	--
Participants included in the research (n = 63)	60 (51)	13–292	--
Participants included in brainstorming (idea generation) (n = 69)	66 (97)	6–736	--
Participants included in sorting (clustering task) (n = 73)	43 (40)	4–271	--
Participants included in ranking (rating task) (n = 68)	53 (55)	13–271	--
Statements generated by participants (n = 56)	331 (437)	45–2704	--
Statements used for sorting and ranting	94 (65)	14–455 ^1^	--
Clusters in the final concept map	7 (2)	3–15	--
Geographical region			
North America	--	--	31 (41)
Europe	--	--	19 (25)
Australasia	--	--	12 (16)
Asia	--	--	7 (9)
Africa	--	--	3 (4)
South America	--	--	1 (1)
Global	--	--	2 (3)
Field of research			
Public health	--	--	25 (33)
Occupational health	--	--	11 (15)
Rehabilitation	--	--	9 (12)
Health policy	--	--	8 (11)
Sexual and reproductive health	--	--	7 (9)
HIV Aids	--	--	6 (8)
Audiology	--	--	5 (7)
Mental health	--	--	4 (5)
Year of publication			
2021 ^2^	--	--	24 (32)
2020	--	--	25 (33)
2019	--	--	26 (35)
Use of any reporting guideline			
Yes	--	--	1 (1)
No	--	--	74 (99)
Availability of peer-review report			
Yes	--	--	7 (9)
No	--	--	68 (91)
Study pre-registration			
Yes	--	--	2 (3)
No	--	--	73 (97)
Publication of study protocol			
Yes	--	--	2 (3)
No	--	--	73 (97
Use of a reporting guideline			
Yes	--	--	1 (1)
No	--	--	74 (99)

^1^ Five studies reported multiple concept maps. ^2^ Indexed in database up to 7 June 2021.

**Table 2:** Frequency and percentage of reporting of the data items in included studies.

S. No.	Reporting Item	Component	Reported	Not Reported	Not Applicable	Percentage Reported ^1^
1	Concept mapping was stated in the study title	Title and abstract	49	26	0	65
2	Concept mapping was reported as a methodology in the abstract	Title and abstract	71	4	0	95
3	Rationale (background information) of study was provided in the abstract	Title and abstract	69	6	0	92
4	Focus question/prompt was reported in the abstract	Title and abstract	16	59	0	21
5	Stakeholders who participated in the study were identified in the abstract	Title and abstract	68	7	0	91
6	Information on the phases of concept mapping was provided in the abstract	Title and abstract	21	54	0	28
7	Study site was reported in the abstract	Title and abstract	20	55	0	27
8	Number of participants in the study was provided in the abstract	Title and abstract	63	12	0	84
9	Information on total number of statements generated in the study was provided in the abstract	Title and abstract	48	27	0	64
10	Number of clusters in the concept map was reported in the abstract	Title and abstract	64	11	0	85
11	Label for all clusters was provided in the abstract	Title and abstract	40	35	0	53
12	Concept mapping software used in the study was stated in the abstract	Title and abstract	7	68	0	9
13	Rationale for the study was explained	Background	70	5	0	93
14	Rationale/justification for concept mapping as a study design was provided	Background	41	34	0	55
15	A clear aim/objective of the study was reported	Background	67	8	0	89
16	The development of the focus prompt was elaborated	Methods—preparation	19	49	7	28
17	Involvement of the stakeholders in the development of focus prompt was reported	Methods—preparation	15	53	7	22
18	Focus prompt used in the study was stated	Methods—preparation	67	1	7	99
19	All the stakeholder groups were identified in the manuscript	Methods—preparation	72	3	0	96
20	Rationale for the stakeholder groups was provided	Methods—preparation	17	58	0	23
21	Participant recruitment was elaborated	Methods—preparation	63	12	0	84
22	Inclusion and exclusion criteria were provided	Methods—preparation	35	40	0	47
23	Data collection period was reported in the manuscript	Methods—preparation	35	40	0	47
24	The process of idea generation was outlined	Methods—idea generation	66	9	0	88
25	Rationale was provided for the number of participants in the idea generation phase	Methods—idea generation	25	48	2	34
26	Information was provided on how brainstorming session was conducted (face-to-face, remote, or both)	Methods—idea generation	71	2	2	97
27	The process of idea synthesis (statement reduction) was detailed	Methods—idea generation	54	20	1	73
28	Involvement of stakeholders in idea synthesis was reported	Methods—idea generation	6	68	1	8
29	Rationale was provided for the number of participants engaged in structuring the statements	Methods—structuring the statements	22	53	0	29
30	Instructions for structuring the statements was reported	Methods—structuring the statements	52	23	0	69
31	Information on how statements were structured (face-to-face, remote, or both) was reported	Methods—structuring the statements	70	5	0	93
32	Web application/software used to structure the statements remotely was reported	Methods—structuring the statements	38	12	25	76
33	Information was provided on the duration of structuring of the statements	Methods—structuring the statements	11	44	20	20
34	Information was provided on the number of prioritization task and type of Likert scale	Methods—structuring the statements	68	0	7	100
35	Name of the software used for data analysis was reported	Methods—data analysis	70	5	0	93
36	Authors outline the steps (statistical procedures) involved in the analysis of concept mapping data	Methods—data analysis	73	2	0	97
37	Information was provided on how cluster solution was identified	Methods—data analysis	49	26	0	65
38	The process of providing cluster labels was reported	Methods—data analysis	61	14	0	81
39	Information was provided on who interpreted the data	Methods—data analysis	67	8	0	89
40	Study participants and/or stakeholders were engaged in data interpretation	Methods—data analysis	65	10	0	87
41	Name of the review board providing ethics approval was mentioned	Additional information	66	9	0	88
42	Authors reported the ethics approval number	Additional information	34	41	0	45
43	The process of obtaining consent from participants was reported	Additional information	55	20	0	73
44	Information on participant reimbursement was provided	Additional information	26	49	0	35
45	Authors reported the total number of participants in the study	Results—participants	50	25	0	67
46	Flow of participants through the different phases of concept mapping was provided	Results—participants	61	14	0	81
47	Sample size for idea generation was reported	Results—participants	68	5	1	93
48	Participant response rate for idea generation was stated	Results—participants	22	51	1	30
49	Number of participants who structured the statements was reported	Results—participants	74	1	0	99
50	Response rate was provided for the statement structuring phase of concept mapping	Results—participants	33	42	0	44
51	Demographic characteristics were reported for all stakeholder groups	Results—participants	68	7	0	91
52	Number (total) of statements generated by the participants was reported	Results—statements	55	19	0	74
53	The number of statements used for structuring phase was reported	Results—statements	72	3	0	96
54	Number of statements beyond those generated by participants was reported	Results—statements	11	64	0	15
55	List of statements used to generate the concept map was provided	Results—statements	63	12	0	84
56	Information was provided on the most and least important statements	Results—statements	20	48	5	29
57	Statements were classified on the basis of a go-zone graph	Results—statements	28	40	5	41
58	Number of clusters generated by the participants (example, mean) was reported	Results—clusters	18	57	0	24
59	The number of cluster solutions considered for interpretation was reported	Results—clusters	34	41	0	45
60	All clusters were identified in the report	Results—clusters	75	0	0	100
61	Authors provided characteristics of the clusters identified in the study	Results—clusters	44	31	0	59
62	The most and least important clusters were reported	Results—clusters	51	17	5	75
63	Authors report the cluster bridging value	Results—clusters	11	64	0	15
64	Information was provided on the stress value and its significance	Results—clusters	51	24	0	68
65	Information was provided on the number of statements in each cluster	Results—clusters	28	47	0	37
66	A ladder graph was computed to report prioritization between stakeholder groups or prioritization tasks	Results—clusters	28	40	5	41
67	Authors discussed the relevance of the study results	Discussion	74	1	0	99
68	A summary of findings from the study was provided	Discussion	62	13	0	83
69	The possible use of the results from the study was reported	Discussion	41	34	0	55
70	A discussion was provided on the limitations of the study	Limitations	71	4	0	95
71	Study was pre-registered, or protocol was published before results	Registration and protocol	3	72	0	4

^1^ The denominator for percentage reported is sum of ‘yes’ and ‘no’.

## Data Availability

Data extracted from the studies included in the review can be assessed in the Supplementary Documents provided with the manuscript.

## Supplementary Materials

The following supporting information can be downloaded at: https://www.mdpi.com/article/10.3390/mps6050101/s1, Supplementary Document S1, PRISMA checklist; Supplementary Document S2, Complete search strategy; Supplementary Document S3, Pilot data extraction tool; Supplementary Document S4, Final data extraction tool; Supplementary Document S5, List of 259 included studies; Supplementary Document S6, List of 88 studies; Supplementary Document S7, List of 13 excluded studies; and Supplementary Document S8, List of 75 studies for data extraction.

## Review Registration

This review was registered prospectively with the Open Science Framework (osf.io/h54k6/).
