# Supplementary material for: A Systematic Review to Inform the Development of a Reporting Guideline for Concept Mapping Research
Source: Methods Protoc. 2023 Oct 17;6(5):101. doi: 10.3390/mps6050101 (PMC10609252; doi:10.3390/mps6050101)
Supplement: Supplementary file 1 [file mps-06-00101-s001.zip › Supplementary document 2_Complete search strategy.pdf]

Additional file 2: Search strategy

Medline

1. Concept mapping study.mp.
2. concept map*.mp.
3. concept mapping.mp.
4. structured conceptualization.mp.
5. 1 or 2 or 3 or 4
6. Ariadne.mp.
7. concept systems.mp.
8. 6 or 7
9. 5 or 8

PsycInfo

1. Concept mapping study.mp.
2. concept map*.mp.
3. concept mapping.mp.
4. structured conceptualization.mp.
5. 1 or 2 or 3 or 4
6. Ariadne.mp.
7. concept systems.mp.
8. 6 or 7
9. 5 or 8

CINAHL

S1 "concept mapping study"
S2 "concept map*"
S3 "concept mapping"
S4 "structured conceptualization"
S5 S1 OR S2 OR S3 OR S4
S6 Ariadne
S7 "concept systems"
S8 S6 OR S7
S9 S5 OR S8
