# Supplementary material for: A Systematic Review to Inform the Development of a Reporting Guideline for Concept Mapping Research
Source: Methods Protoc. 2023 Oct 17;6(5):101. doi: 10.3390/mps6050101 (PMC10609252; doi:10.3390/mps6050101)
Supplement: Supplementary file 1 [file mps-06-00101-s001.zip › Supplementary document 5_List of 258 included studies.pdf]

List of included studies

Aarons, G. A., Reeder, K., Sam-Agudu, N. A., Vorkoper, S., & Sturke, R. (2021). Implementation determinants and mechanisms for the prevention and treatment of adolescent HIV in sub-Saharan Africa: concept mapping of the NIH Fogarty International Center Adolescent HIV Implementation Science Alliance (AHISA) initiative. *Implementation science communications, 2*(1), 53. https://doi.org/https://dx.doi.org/10.1186/s43058-021-00156-3

Aarons, G. A., Sommerfeld, D. H., Chi, B. H., Ezeanolue, E. E., Sturke, R., Guay, L., & Siberry, G. K. (2016). Concept Mapping of PMTCT Implementation Challenges and Solutions Across 6 sub-Saharan African Countries in the NIH-PEPFAR PMTCT Implementation Science Alliance. *Journal of acquired immune deficiency syndromes (1999), 72 Suppl 2*, S202-206. https://doi.org/https://dx.doi.org/10.1097/QAI.0000000000001064

Ageberg, E., Bunke, S., Lucander, K., Nilsen, P., & Donaldson, A. (2019). Facilitators to support the implementation of injury prevention training in youth handball: A concept mapping approach. *Scandinavian journal of medicine & science in sports, 29*(2), 275-285. https://doi.org/https://dx.doi.org/10.1111/sms.13323

Ahmad, F., Mahmood, S., Pietkiewicz, I., McDonald, L., & Ginsburg, O. (2012). Concept mapping with South Asian immigrant women: Barriers to mammography and solutions. *Journal of Immigrant and Minority Health, 14*(2), 242-250. https://doi.org/http://dx.doi.org/10.1007/s10903-011-9472-7

Ahmad, F., Norman, C., & O'Campo, P. (2012). What is needed to implement a computer-assisted health risk assessment tool? An exploratory concept mapping study. *BMC medical informatics and decision making, 12*, 149. https://doi.org/https://dx.doi.org/10.1186/1472-6947-12-149

Anderson, L. A., Gwaltney, M. K., Sundra, D. L., Brownson, R. C., Kane, M., Cross, A. W., Mack, R., Jr., Schwartz, R., Sims, T., & Carol, W. R. (2006). Using concept mapping to develop a logic model for the Prevention Research Centers Program. *Preventing chronic disease, 3*(1), A06. http://ovidsp.ovid.com/ovidweb.cgi?T=JS&PAGE=reference&D=med6&NEWS=N&AN=16356359

Antoniou, T., Mishra, S., Matheson, F., Smith-Merrill, D., Challacombe, L., Rowe, J., DiCenso, A. M., Kouyoumdjian, F. G., Wobeser, W., Kendall, C., Loutfy, M., Tsang, J., Kanee, L., & Strike, C. (2019). Using concept mapping to inform the development of a transitional reintegration intervention program for formerly incarcerated people with HIV. *BMC health services research, 19*(1), 761. https://doi.org/https://dx.doi.org/10.1186/s12913-019-4595-y

Ast, R. S., Banyard, V. L., Burnham, J., & Edwards, K. M. (2021). Community conversations on relationship violence: Town variations in prevention perceptions through concept mapping. *American Journal of Community Psychology*, No-Specified. https://doi.org/http://dx.doi.org/10.1002/ajcp.12488

Ayala, E. E., & Almond, A. L. (2018). Self-care of women enrolled in health service psychology programs: A concept mapping approach. *Professional Psychology: Research & Practice, 49*(3), 177-184. https://doi.org/10.1037/pro0000190

Baars, E. W., van der Hart, O., Nijenhuis, E. R. S., Chu, J. A., Glas, G., & Draijer, N. A. (2011). Predicting stabilizing treatment outcomes for complex posttraumatic stress disorder and dissociative identity disorder: An expertise-based prognostic model. *Journal of Trauma & Dissociation, 12*(1), 67-87. https://doi.org/http://dx.doi.org/10.1080/15299732.2010.514846

Barnert, E. S., Coller, R. J., Nelson, B. B., Thompson, L. R., Klitzner, T. S., Szilagyi, M., Breck, A. M., & Chung, P. J. (2018). A Healthy Life for a Child With Medical Complexity: 10 Domains for Conceptualizing Health. *Pediatrics, 142*(3). https://doi.org/https://dx.doi.org/10.1542/peds.2018-0779

Baskin, M. L., Dulin-Keita, A., Thind, H., & Godsey, E. A. (2015). Social and cultural environment factors influencing physical activity among African-American adolescents. *Journal of Adolescent Health, 56*(5), 536-542. https://doi.org/http://dx.doi.org/10.1016/j.jadohealth.2015.01.012

Batterham, R., Southern, D., Appleby, N., Elsworth, G., Fabris, S., Dunt, D., & Young, D. (2002). Construction of a GP integration model. *Social science & medicine (1982), 54*(8), 1225-1241. http://ovidsp.ovid.com/ovidweb.cgi?T=JS&PAGE=reference&D=med4&NEWS=N&AN=11989959

Beckers, T., Koekkoek, B., Hutschemaekers, G., & Tiemens, B. (2018). Potential predictive factors for successful referral from specialist mental-health services to less intensive treatment: A concept mapping study. *PLoS ONE, 13*(6), e0199668. https://doi.org/https://dx.doi.org/10.1371/journal.pone.0199668

Bedi, R. P., & Richards, M. A. (2011). What a man wants: The male perspective on therapeutic alliance formation. *Special Issue: Evidence-Based Psychotherapy Relationships II, 48*(4), 381-390. https://doi.org/http://dx.doi.org/10.1037/a0022424

Behar, L. B., & Hydaker, W. M. (2009). Defining community readiness for the implementation of a system of care. *Administration and Policy in Mental Health and*

Mental Health Services Research, 36(6), 381-392. https://doi.org/http://dx.doi.org/10.1007/s10488-009-0227-x

Belaid, L., Dagenais, C., Moha, M., & Ridde, V. (2017). Understanding the factors affecting the attraction and retention of health professionals in rural and remote areas: a mixed-method study in Niger. *Human resources for health, 15*(1), 60. https://doi.org/https://dx.doi.org/10.1186/s12960-017-0227-y

Belmon, L. S., Busch, V., van Stralen, M. M., Stijnman, D. P. M., Hidding, L. M., Harmsen, I. A., & Chinapaw, M. J. M. (2020). Child and Parent Perceived Determinants of Children's Inadequate Sleep Health. A Concept Mapping Study. *International journal of environmental research and public health, 17*(5). https://doi.org/https://dx.doi.org/10.3390/ijerph17051583

Bennett, R. J., Barr, C., Montano, J., Eikelboom, R. H., Saunders, G. H., Pronk, M., Preminger, J. E., Ferguson, M., Weinstein, B., Heffernan, E., van Leeuwen, L., Hickson, L., Timmer, B. H. B., Singh, G., Gerace, D., Cortis, A., & Bellekom, S. R. (2021). Identifying the approaches used by audiologists to address the psychosocial needs of their adult clients. *International Journal of Audiology, 60*(2), 104-114. https://doi.org/https://dx.doi.org/10.1080/14992027.2020.1817995

Bennett, R. J., Fletcher, S., Conway, N., & Barr, C. (2020). The role of the general practitioner in managing age-related hearing loss: perspectives of general practitioners, patients and practice staff. *BMC family practice, 21*(1), 87. https://doi.org/https://dx.doi.org/10.1186/s12875-020-01157-2

Bennett, R. J., Laplante-Levesque, A., Meyer, C. J., & Eikelboom, R. H. (2018). Exploring Hearing Aid Problems: Perspectives of Hearing Aid Owners and Clinicians. *Ear and hearing, 39*(1), 172-187. https://doi.org/https://dx.doi.org/10.1097/AUD.0000000000000477

Bennett, R. J., Meyer, C. J., & Eikelboom, R. H. (2019). How do hearing aid owners acquire hearing aid management skills? *Journal of the American Academy of Audiology, 30*(6), 516-532. http://ovidsp.ovid.com/ovidweb.cgi?T=JS&PAGE=reference&D=psyc17&NEWS=N&AN=2019-46922-007

Bennett, R. J., Meyer, C. J., Eikelboom, R. H., & Atlas, M. D. (2018). Investigating the knowledge, skills, and tasks required for hearing aid management: Perspectives of clinicians and hearing aid owners. *American Journal of Audiology, 27*(1), 67-84. https://doi.org/http://dx.doi.org/10.1044/2017_AJA-17-0059

Brintnell, E. S., Sommer, R. W., Kuncoro, B., Setiawan, G. P., & Bailey, P. (2013). The expression of depression among Javanese patients with major depressive disorder: A concept mapping study. *Transcultural Psychiatry, 50*(4), 579-598. https://doi.org/http://dx.doi.org/10.1177/1363461513501709

Brons, A., Braam, K., Timmerman, A., Broekema, A., Visser, B., van Ewijk, B., Terheggen-Lagro, S., Rutjes, N., van Leersum, H., Engelbert, R., Krose, B., Chinapaw, M., & Altenburg, T. (2019). Promoting Factors for Physical Activity in Children with Asthma Explored through Concept Mapping. *International journal of environmental research and public health, 16*(22). https://doi.org/https://dx.doi.org/10.3390/ijerph16224467

Brown, E., Topping, A., & Cheston, R. (2019). What are the barriers to accessing psychological therapy in Qatar: A concept mapping study. *Counselling & Psychotherapy Research, 19*(4), 441-454. https://doi.org/10.1002/capr.12252

Brown, J., & Anderberg, A. (2007). Fostering children with disabilities: A concept map of parent needs. *Children and Youth Services Review, 29*(9), 1235-1248. https://doi.org/http://dx.doi.org/10.1016/j.childyouth.2007.05.009

Brown, J., Kapasi, A., Nowicki, E., & Cleversey, K. (2019). Expectations of youth with a fetal alcohol spectrum disorder in adulthood: Caregiver perspectives. *Journal on Developmental Disabilities, 24*(2), 29-42. http://ovidsp.ovid.com/ovidweb.cgi?T=JS&PAGE=reference&D=psyc17&NEWS=N&AN=2020-61329-004

Brown, J., Wiendels, S., Eyre, V., & Ali, A. (2019). Social justice competencies for counselling and psychotherapy: Perceptions of experienced practitioners and implications for contemporary practice. *Counselling & Psychotherapy Research, 19*(4), 533-543. https://doi.org/http://dx.doi.org/10.1002/capr.12247

Brown, J. D., Bednar, L. M., Sigvaldason, N., & Anderberg, A. (2007). Causes of placement breakdown for foster children affected by alcohol. *Child & Adolescent Social Work Journal, 24*(4), 313-332. https://doi.org/http://dx.doi.org/10.1007/s10560-007-0086-9

Bruder, A. M., Crossley, K. M., Mosler, A. B., Patterson, B., Haberfield, M., & Donaldson, A. (2020). Co-creation of a sport-specific anterior cruciate ligament injury risk reduction program for women: A concept mapping approach. *Journal of science and medicine in sport, 23*(4), 353-360. https://doi.org/https://dx.doi.org/10.1016/j.jsams.2019.10.019

Buitenweg, D. C., Bongers, I. L., van de Mheen, D., van Oers, H. A. M., Van Nieuwenhuizen, & Chijs, A. (2018). Worth a thousand words? Visual concept mapping of the quality of life of people with severe mental health problems. *International Journal of Methods in Psychiatric Research, 27*(3), 1-10. https://doi.org/http://dx.doi.org/10.1002/mpr.1721

Burke, J., O'Campo, P., Salmon, C., Walker, R., & Barrett, B. (2009). Pathways connecting neighborhood influences and mental well-being: Socioeconomic position and gender differences. *Social Science & Medicine, 68*(7), 1294-1304. https://doi.org/http://dx.doi.org/10.1016/j.socscimed.2009.01.015

Busija, L., Cinelli, R., Toombs, M. R., Easton, C., Hampton, R., Holdsworth, K., Macleod, A., Nicholson, G. C., Nasir, B. F., Sanders, K. M., & McCabe, M. P. (2020). The Role of Elders in the Wellbeing of a Contemporary Australian Indigenous Community. *Gerontologist, 60*(3), 513-524. https://doi.org/10.1093/geront/gny140

Caarls, P. J., van Schijndel, M. A., Berk, G. v. d., Boenink, A. D., Boerman, D., Lijmer, J. G., Honig, A., Terra, M., Thijs, A., Verwey, B., Waarde, J. A. v., Wijngaarden, J. v., Busschbach, J. J. v., & Group, M. P. U. S. (2019). Factors influencing the admission decision for Medical Psychiatry Units: A concept mapping approach. *PLoS ONE, 14*(9), e0221807. https://doi.org/https://dx.doi.org/10.1371/journal.pone.0221807

Cardwell, R., McKenna, L., Davis, J., & Gray, R. (2021). How is clinical credibility defined in nursing? A concept mapping study. *Journal of Clinical Nursing*, No-Specified. https://doi.org/http://dx.doi.org/10.1111/jocn.15572

Chakraborty, A., Howard, N. J., Daniel, M., Chong, A., Slavin, N., Brown, A., & Cargo, M. (2021). Prioritizing Built Environmental Factors to Tackle Chronic and Infectious Diseases in Remote Northern Territory (NT) Communities of Australia: A Concept Mapping Study. *International journal of environmental research and public health, 18*(10). https://doi.org/https://dx.doi.org/10.3390/ijerph18105178

Chan, C. W. H., Choi, K. C., Chan, H. Y. L., Wong, M. M. H., Ling, G. C. C., Chow, K. M., Chow, A. Y. M., Lo, R., Sham, M. M. K., & Alano, B. (2019). Unfolding and displaying the influencing factors of advance directives from the stakeholder's perspective: A concept mapping approach. *Journal of Advanced Nursing, 75*(7), 1549-1562. https://doi.org/http://dx.doi.org/10.1111/jan.14017

Chiauzzi, E., Trudeau, K. J., Zacharoff, K., & Bond, K. (2011). Identifying primary care skills and competencies in opioid risk management. *The Journal of continuing education in the health professions, 31*(4), 231-240. https://doi.org/https://dx.doi.org/10.1002/chp.20135

Cho, H., Seo, Y. S., & Bedi, C. (2017). Korean counselors' perceptions of the real relationship in counseling process. *Asia Pacific Education Review, 18*(1), 135-146. https://doi.org/http://dx.doi.org/10.1007/s12564-016-9450-9

Chow, K. M., Chan, C. W. H., Choi, K. C., White, I. D., Siu, K. Y., & Sin, W. H. (2021). A practice model of sexuality nursing care: a concept mapping approach. *Supportive care in cancer : official journal of the Multinational Association of Supportive Care in Cancer, 29*(3), 1663-1673. https://doi.org/https://dx.doi.org/10.1007/s00520-020-05660-1

Cleversey, K., Brown, J., Kapasi, A., & Bailey, B. (2017). Educating adolescents with fetal alcohol spectrum disorder: Caregiver support needs. *Journal of Child and Family Studies, 26*(10), 2843-2851. https://doi.org/http://dx.doi.org/10.1007/s10826-017-0776-7

Cook-Cottone, C., Giambrone, C., Klein, J., & Abadi, A. (2018). Yoga for Kenyan children: Concept-mapping with multidimensional scaling and hierarchical cluster analysis. *International Journal of School & Educational Psychology, 6*(3), 151-164. https://doi.org/http://dx.doi.org/10.1080/21683603.2017.1302852

Cook, K. A., & Bergeron, K. (2020). Palliative care for young adults with life-limiting conditions: public health recommendations. *BMJ supportive & palliative care*. https://doi.org/https://dx.doi.org/10.1136/bmjspcare-2019-002042

D'Alonzo, K. T., Vilaro, F. M., Joseph, M. E., Oyeneye, V., Garsman, L., Rosas, S. R., Castaneda, M., & Vivar, M. (2020). Using Concept Mapping within a Community-Academic Partnership to Examine Obesity among Mexican Immigrants. *Progress in community health partnerships : research, education, and action, 14*(2), 173-185. https://doi.org/https://dx.doi.org/10.1353/cpr.2020.0016

Dagenais, C., Degroote, S., Otmani Del Barrio, M., Bermudez-Tamayo, C., & Ridde, V. (2018). Establishing research priorities in prevention and control of vector-borne diseases in urban areas: a collaborative process. *Infectious diseases of poverty, 7*(1), 85. https://doi.org/https://dx.doi.org/10.1186/s40249-018-0463-y

Daoud, N., Jung, Y. E., Muhammad, A. S., Weinstein, R., Qaadny, A., Ghattas, F., Khatib, M., Grotto, I., & Abdullah, A.-s. (2018). Facilitators and barriers to smoking cessation among minority men using the behavioral-ecological model and Behavior Change Wheel: A concept mapping study. *PLoS ONE, 13*(10). https://doi.org/http://dx.doi.org/10.1371/journal.pone.0204657

Daughtry, D., Kunkel, M. A., & Athens, B. (1993). Experience of depression in college students: A concept map. *Journal of Counseling Psychology, 40*(3), 316-323. https://doi.org/http://dx.doi.org/10.1037/0022-0167.40.3.316

Davis, T. S., Saltzburg, S., Locke, C. R., & Avery, B. (2009). Supporting the emotional and psychological well being of sexual minority youth: Youth ideas for action. *Children and Youth Services Review, 31*(9), 1030-1041. https://doi.org/http://dx.doi.org/10.1016/j.childyouth.2009.05.003

Dawson, A. P., Cargo, M., Stewart, H., Chong, A., & Daniel, M. (2013). Identifying multi-level culturally appropriate smoking cessation strategies for Aboriginal health staff: A concept mapping approach. *Health Education Research, 28*(1), 31-45. https://doi.org/http://dx.doi.org/10.1093/her/cys111

de Boer, M. E., Depla, M. F. I. A., Frederiks, B. J. M., Negenman, A. A., Habraken, J. M., van Randeraad-van der Zee, C. H., Embregts, P. J. C. M., & Hertogh, C. M. P. M. (2019). Involuntary care-Capturing the experience of people with dementia in nursing homes. A concept mapping study. *Aging & Mental Health, 23*(4), 498-506. https://doi.org/http://dx.doi.org/10.1080/13607863.2018.1428934

de Kok, M., Scholte, R. W., Sixma, H. J., van der Weijden, T., Spijkers, K. F., van de Velde, C. J., Roukema, J. A., van der Ent, F. W., Bell, A. V., & von Meyenfeldt, M. F. (2007). The patient's perspective of the quality of breast cancer care. The development of an instrument to measure quality of care through focus groups and concept mapping with breast cancer patients. *European Journal of Cancer, 43*(8), 1257-1264. https://doi.org/10.1016/j.ejca.2007.03.012

de Ridder, D., Depla, M., Severens, P., & Malsch, M. (1997). Beliefs on coping with illness: a consumer's perspective. *Social science & medicine (1982), 44*(5), 553-559. http://ovidsp.ovid.com/ovidweb.cgi?T=JS&PAGE=reference&D=med4&NEWS=N&AN=9032823

De Ridder, D. T., Schreurs, K. M., & Bensing, J. M. (1998). Adaptive tasks, coping and quality of life of chronically ill patients: the cases of Parkinson's disease and chronic fatigue syndrome. *Journal of Health Psychology, 3*(1), 87-101. https://doi.org/https://dx.doi.org/10.1177/135910539800300107

de Snoo-Trimp, J. C., Molewijk, B., & de Vet, H. C. W. (2018). Defining and categorizing outcomes of Moral Case Deliberation (MCD): Concept mapping with experienced MCD participants. *BMC Medical Ethics, 19*. https://doi.org/http://dx.doi.org/10.1186/s12910-018-0324-z

de Vries, G., Koeter, M. W. J., Nabitz, U., Hees, H. L., & Schene, A. H. (2012). Return to work after sick leave due to depression; A conceptual analysis based on perspectives of patients, supervisors and occupational physicians. *Journal of Affective Disorders, 136*(3), 1017-1026. https://doi.org/http://dx.doi.org/10.1016/j.jad.2011.06.035

Donaldson, A., Callaghan, A., Bizzini, M., Jowett, A., Keyzer, P., & Nicholson, M. (2019). A concept mapping approach to identifying the barriers to implementing an evidence-based sports injury prevention programme. *Injury prevention : journal of the International Society for Child and Adolescent Injury Prevention, 25*(4), 244-251. https://doi.org/https://dx.doi.org/10.1136/injuryprev-2017-042639

Donaldson, A., Reimers, J. L., Brophy, K. T., & Nicholson, M. (2019). Barriers to rejecting junk food sponsorship in sport-a formative evaluation using concept mapping. *Public Health, 166*, 1-9. https://doi.org/https://dx.doi.org/10.1016/j.puhe.2018.09.021

Donnelly, J. P., Donnelly, K., & Grohman, K. K. (2005). A multi-perspective concept mapping study of problems associated with traumatic brain injury. *Brain Injury, 19*(13), 1077-1085. http://ovidsp.ovid.com/ovidweb.cgi?T=JS&PAGE=reference&D=med6&NEWS=N&AN=16286321

Donnelly, K. Z., Donnelly, J. P., & Grohman, K. J. (2000). Cognitive, emotional, and behavioral problems associated with traumatic brain injury: A concept map of patient, family, and provider perspectives. *Special Issue: 9th Annual Rotman Research Inst Conference "Traumatic brain injury: Diagnosis, outcome, & rehabilitation" March 24-26, 1999., 44*(1), 21-25. https://doi.org/http://dx.doi.org/10.1016/S0278-2626%2820%2930181-0

Dopp, A. R., Parisi, K. E., Munson, S. A., & Lyon, A. R. (2020). Aligning implementation and user-centered design strategies to enhance the impact of health services: results from a concept mapping study. *Implementation science communications, 1*, 17. https://doi.org/https://dx.doi.org/10.1186/s43058-020-00020-w

Doty, A. M. B., Powell, R. E., Carr, B. G., Nelson, D. B., & Rising, K. L. (2018). Identification of Approaches to Improve Patient Trust in Health Systems: A Group Concept Mapping Study. *Journal of healthcare management / American College of Healthcare Executives, 63*(5), e116-e129. https://doi.org/https://dx.doi.org/10.1097/JHM-D-17-00037

Du Bois, S. N., Guy, A. A., Manser, K. A., Thomas, N. N., Noble, S., Lewis, R., Toles, J., Spivey, C., Khan, H., & Tully, T. (2020). Developing prepare2thrive, a community-based intervention targeting treatment engagement among african american individuals living with hiv and serious mental illness. *AIDS Care*, No-Specified. https://doi.org/http://dx.doi.org/10.1080/09540121.2020.1717420

Dulin, A. J., Earnshaw, V. A., Dale, S. K., Carey, M. P., Fava, J. L., Wilson-Barthes, M., Mugavero, M. J., Dougherty-Sheff, S., Johnson, B., Napravnik, S., & Howe, C. J.

(2021). A Concept Mapping Study to Understand Multilevel Resilience Resources Among African American/Black Adults Living with HIV in the Southern United States. *AIDS and Behavior, 25*(3), 773-786. https://doi.org/https://dx.doi.org/10.1007/s10461-020-03042-6

Dulin Keita, A., Clay, O., Whittaker, S., Hannon, L., Adams, I. K., Rogers, M., & Gans, K. (2015). The influence of HOPE VI neighborhood revitalization on neighborhood-based physical activity: A mixed-methods approach. *Social Science & Medicine, 139*, 90-99. https://doi.org/http://dx.doi.org/10.1016/j.socscimed.2015.06.002

Dulin Keita, A., Hannon, L., Buys, D., Casazza, K., & Clay, O. (2016). Surrounding Community Residents' Expectations of HOPE VI for Their Community, Health and Physical Activity. *Journal of community practice, 24*(1), 18-37. https://doi.org/10.1080/10705422.2015.1129005

Dunlop, S., Lewis, N., Richardson, R., Thomas, S., Devonald-Morris, M., Pontin, D., & Wallace, C. (2020). Using group concept mapping to explore the complexities of managing children's care. *Nurse researcher*. https://doi.org/https://dx.doi.org/10.7748/nr.2020.e1696

Ebrahimi-Madiseh, A., Eikelboom, R. H., Bennett, R. J., Upson, G. S., Friedland, P. L., Swanepoel, D. W., Psarros, C., Lai, W. K., & Atlas, M. D. (2020). What Influences Decision-Making for Cochlear Implantation in Adults? Exploring Barriers and Drivers From a Multistakeholder Perspective. *Ear and hearing, 41*(6), 1752-1763. https://doi.org/https://dx.doi.org/10.1097/AUD.0000000000000895

Eigeland, J. A., Jones, L., Sheeran, N., & Moffitt, R. L. (2021). Critical physician behaviors in the formation of a good physician-patient relationship: Concept mapping the perspective of patients with chronic conditions. *Patient Education and Counseling*, No-Specified. https://doi.org/http://dx.doi.org/10.1016/j.pec.2021.04.035

Elsman, E. B. M., van Rens, G. H. M. B., & van Nispen, R. M. A. (2017). Impact of visual impairment on the lives of young adults in the Netherlands: A concept-mapping approach. *Disability and Rehabilitation: An International, Multidisciplinary Journal, 39*(26), 2607-2618. https://doi.org/http://dx.doi.org/10.1080/09638288.2016.1236408

Esmaeili, N., & Bamdad Soofi, J. (2021). Expounding the knowledge conversion processes within the occupational safety and health management system (OSH-MS) using concept mapping. *International journal of occupational safety and ergonomics : JOSE*, 1-16. https://doi.org/https://dx.doi.org/10.1080/10803548.2020.1853957

Ettinger, A. K., Ray, K. N., Burke, J. G., Thompson, J., Navratil, J., Chavis, V., Cole, S., Jenks, T., & Miller, E. (2021). A Community Partnered Approach for Defining Child and Youth Thriving. *Academic pediatrics, 21*(1), 53-62. https://doi.org/https://dx.doi.org/10.1016/j.acap.2020.04.011

Felx, A., Kane, M., Corbiere, M., & Lesage, A. (2020). Using Group Concept Mapping to Develop a Conceptual Model of Housing and Community-Based Residential Settings for Adults With Severe Mental Illness. *Frontiers in psychiatry, 11*, 430. https://doi.org/https://dx.doi.org/10.3389/fpsyt.2020.00430

Fitzpatrick, S. J., & Zizzi, S. J. (2014). Using Concept Mapping to Identify Action Steps for Physical Activity Promotion in Cancer Treatment. *American Journal of Health Education, 45*(1), 20-28. https://doi.org/10.1080/19325037.2013.852999

Forsdike, K., Donaldson, A., & Seal, E. (2020). Responding to Violence Against Women in Sport: Challenges Facing Sport Organizations in Victoria, Australia. *Research quarterly for exercise and sport*, 1-16. https://doi.org/https://dx.doi.org/10.1080/02701367.2020.1844857

Frye, V., Paul, M. M., Todd, M.-J., Lewis, V., Cupid, M., Coleman, J., Salmon, C., & O'Campo, P. (2012). Informal social control of intimate partner violence against women: Results from a concept mapping study of urban neighborhoods. *Journal of Community Psychology, 40*(7), 828-844. https://doi.org/http://dx.doi.org/10.1002/jcop.21493

Gausman, J., Lloyd, D., Kallon, T., Subramanian, S. V., Langer, A., & Austin, S. B. (2019). Clustered risk: An ecological understanding of sexual activity among adolescent boys and girls in two urban slums in Monrovia, Liberia. *Social Science & Medicine, 224*, 106-115. https://doi.org/http://dx.doi.org/10.1016/j.socscimed.2019.02.010

Gausman, J., Othman, A., Daas, I., Hamad, I., Dabobe, M., & Langer, A. (2021). How Jordanian and Syrian youth conceptualise their sexual and reproductive health needs: a visual exploration using concept mapping. *Culture, Health & Sexuality, 23*(2), 176-191. https://doi.org/https://dx.doi.org/10.1080/13691058.2019.1698769

Giambrone, C. A., Cook-Cottone, C. P., & Klein, J. E. (2018). The Africa yoga project and well-being: A concept map of students' perceptions. *Applied Psychology: Health and Well-Being, 10*(1), 149-170. https://doi.org/http://dx.doi.org/10.1111/aphw.12124

Glimmerveen, J. C., Brazil, I. A., Bulten, B. H., & Maes, J. H. R. (2018). Uncovering naturalistic rewards and their subjective value in forensic psychiatric patients. *The International Journal of Forensic Mental Health, 17*(2), 154-166. https://doi.org/http://dx.doi.org/10.1080/14999013.2018.1452081

Glista, D., O'Hagan, R., Moodie, S., & Scollie, S. (2021). An examination of clinical uptake factors for remote hearing aid support: a concept mapping study with audiologists. *International Journal of Audiology, 60*(sup1), S13-S22. https://doi.org/https://dx.doi.org/10.1080/14992027.2020.1795281

Green, A. E., & Aarons, G. A. (2011). A comparison of policy and direct practice stakeholder perceptions of factors affecting evidence-based practice implementation using concept mapping. *Implementation science : IS, 6*, 104. https://doi.org/https://dx.doi.org/10.1186/1748-5908-6-104

Green, C., Walkup, J. T., Bostwick, S., & Trochim, W. (2019). Advancing the agenda in pediatric mental health education. *Pediatrics, 144*(3). https://doi.org/http://dx.doi.org/10.1542/peds.2018-2596

Guilcher, S. J. T., Cadel, L., Everall, A. C., Wiese, J. L., Hamilton-Wright, S., Salmon, C. C., & Matheson, F. I. (2020). Factors related to screening for problem gambling among healthcare and social service providers in Ontario, Canada: A concept mapping study. *Health & Social Care in the Community, 28*(3), 791-802. https://doi.org/https://dx.doi.org/10.1111/hsc.12909

Haafkens, J., Blomstedt, Y., Eriksson, M., Becher, H., Ramroth, H., & Kinsman, J. (2014). Training needs for research in health inequities among health and demographic researchers from eight African and Asian countries. *BMC public health, 14*, 1254. https://doi.org/https://dx.doi.org/10.1186/1471-2458-14-1254

Haafkens, J. A., Kopnina, H., Meerman, M. G. M., & van Dijk, F. J. H. (2011). Facilitating job retention for chronically ill employees: perspectives of line managers and human resource managers. *BMC health services research, 11*, 104. https://doi.org/https://dx.doi.org/10.1186/1472-6963-11-104

Hackett, K. L., Deane, K. H. O., Newton, J. L., Deary, V., Bowman, S. J., Rapley, T., & Ng, W.-F. (2018). Mixed-Methods Study Identifying Key Intervention Targets to Improve Participation in Daily Living Activities in Primary Sjogren's Syndrome Patients. *Arthritis care & research, 70*(7), 1064-1073. https://doi.org/https://dx.doi.org/10.1002/acr.23536

Hackett, K. L., Lambson, R. L., Strassheim, V., Gotts, Z., Deary, V., & Newton, J. L. (2016). A concept mapping study evaluating the UK's first NHS generic fatigue clinic. *Health Expectations: An International Journal of Public Participation in Health Care & Health Policy, 19*(5), 1138-1149. https://doi.org/http://dx.doi.org/10.1111/hex.12405

Hammarlund, C. S., Nilsson, M. H., & Hagell, P. (2012). Measuring outcomes in Parkinson's disease: A multi-perspective concept mapping study. *Quality of Life Research: An International Journal of Quality of Life Aspects of Treatment, Care & Rehabilitation, 21*(3), 453-463. https://doi.org/http://dx.doi.org/10.1007/s11136-011-9995-3

Hammarlund, C. S., Nilsson, M. H., Idvall, M., Rosas, S. R., & Hagell, P. (2014). Conceptualizing and prioritizing clinical trial outcomes from the perspectives of people with Parkinson's disease versus health care professionals: A concept mapping study. *Quality of Life Research: An International Journal of Quality of Life Aspects of Treatment, Care & Rehabilitation, 23*(6), 1687-1700. https://doi.org/http://dx.doi.org/10.1007/s11136-013-0614-3

Hanson, H. M., Schiller, C., Winters, M., Sims-Gould, J., Clarke, P., Curran, E., Donaldson, M. G., Pitman, B., Scott, V., McKay, H. A., & Ashe, M. C. (2013). Concept mapping applied to the intersection between older adults' outdoor walking and the built and social environments. *Preventive Medicine: An International Journal Devoted to Practice and Theory, 57*(6), 785-791. https://doi.org/http://dx.doi.org/10.1016/j.ypmed.2013.08.023

Hanzen, G., van Nispen, R. M. A., van der Putten, A. A. J., & Waninge, A. (2017). Participation of adults with visual and severe or profound intellectual disabilities: Definition and operationalization. *Research in Developmental Disabilities, 61*, 95-107. https://doi.org/http://dx.doi.org/10.1016/j.ridd.2016.12.017

Hargett, C. W., Doty, J. P., Hauck, J. N., Webb, A. M., Cook, S. H., Tsipis, N. E., Neumann, J. A., Andolsek, K. M., & Taylor, D. C. (2017). Developing a model for effective leadership in healthcare: a concept mapping approach. *Journal of healthcare leadership, 9*, 69-78. https://doi.org/https://dx.doi.org/10.2147/JHL.S141664

Hargreaves, K., & Crozier, K. (2013). A conceptual understanding of the factors that influence breastfeeding cessation. *Evidence Based Midwifery, 11*(3), 81-87. http://ez.library.latrobe.edu.au/login?url=http://search.ebscohost.com/login.aspx?direct=true&db=cin20&AN=90148987&site=ehost-live&scope=site

Hart, K. M., & Neil, N. (2021). Down syndrome caregivers' support needs: a mixed-method participatory approach. *Journal of intellectual disability research : JIDR, 65*(1), 60-76. https://doi.org/https://dx.doi.org/10.1111/jir.12791

Hatcher, S. S. (2010). Recognizing perspectives on community reentry from offenders with mental illness: Using the Afrocentric framework and concept mapping with adult detainees. *Journal of Offender Rehabilitation, 49*(8), 536-550. https://doi.org/http://dx.doi.org/10.1080/10509674.2010.519649

Heinonen, H., Volin, L., Zevon, M. A., Uutela, A., Barrick, C., & Ruutu, T. (2005). Stress among allogeneic bone marrow transplantation patients. *Patient Education and Counseling, 56*(1), 62-71. https://doi.org/http://dx.doi.org/10.1016/j.pec.2003.12.007

Hidding, L. M., Altenburg, T. M., van Ekris, E., & Chinapaw, M. J. M. (2017). Why Do Children Engage in Sedentary Behavior? Child- and Parent-Perceived Determinants. *International journal of environmental research and public health, 14*(7). https://doi.org/https://dx.doi.org/10.3390/ijerph14070671

Hiler, M., Spindle, T. R., Dick, D., Eissenberg, T., Breland, A., & Soule, E. (2020). Reasons for transition from electronic cigarette use to cigarette smoking among young adult college students. *Journal of Adolescent Health, 66*(1), 56-63. https://doi.org/http://dx.doi.org/10.1016/j.jadohealth.2019.09.003

Houle, A.-A., Besnard, T., Bérubé, A., & Dagenais, C. (2018). Factors that influence parent recruitment into prevention programs in early childhood: A concept map of parents', practitioners', and administrators' points of view. *Children & Youth Services Review, 85*, 127-136. https://doi.org/10.1016/j.childyouth.2017.12.014

Hvidt, N. C., Nielsen, K. T., Korup, A. K., Prinds, C., Hansen, D. G., Viftrup, D. T., Assing Hvidt, E., Hammer, E. R., Falko, E., Locher, F., Boelsbjerg, H. B., Wallin, J. A., Thomsen, K. F., Schroder, K., Moestrup, L., Nissen, R. D., Stewart-Ferrer, S., Stripp, T. K., Steenfeldt, V. O., Sondergaard, J., & Waehrens, E. E. (2020). What is spiritual care? Professional perspectives on the concept of spiritual care identified through group concept mapping. *BMJ open, 10*(12), e042142. https://doi.org/https://dx.doi.org/10.1136/bmjopen-2020-042142

Iris, M., DeBacker, N. A., Benner, R., Hammerman, J., & Ridings, J. (2012). Creating a quality of life assessment measure for residents in long term care. *Journal of the American Medical Directors Association, 13*(5), 438-447. https://doi.org/https://dx.doi.org/10.1016/j.jamda.2011.08.011

Iris, M., Ridings, J. W., & Conrad, K. J. (2010). The development of a conceptual model for understanding elder self-neglect. *The Gerontologist, 50*(3), 303-315. https://doi.org/http://dx.doi.org/10.1093/geront/gnp125

Iwelunmor, J., Blackstone, S., Gyamfi, J., Airhihenbuwa, C., Plange-Rhule, J., Tayo, B., Adanu, R., & Ogedegbe, G. (2015). A Concept Mapping Study of Physicians' Perceptions of Factors Influencing Management and Control of Hypertension in Sub-Saharan Africa. *International journal of hypertension, 2015*, 412804. https://doi.org/https://dx.doi.org/10.1155/2015/412804

Jaynes, C. L., Werman, H. A., & White, L. J. (2013). A blueprint for critical care transport research. *Air medical journal, 32*(1), 30-35. https://doi.org/https://dx.doi.org/10.1016/j.amj.2012.11.001

Jenkins, A. M., Burns, D., Horick, R., Spicer, B., Vaughn, L. M., & Woodward, J. (2021). Adolescents and Young Adults With Spina Bifida Transitioning to Adulthood: A Comprehensive Community-Based Needs Assessment. *Academic pediatrics*. https://doi.org/https://dx.doi.org/10.1016/j.acap.2021.02.002

Johnson, D. B., Quinn, E., Sitaker, M., Ammerman, A., Byker, C., Dean, W., Fleischhacker, S., Kolodinsky, J., Pinard, C., Pitts, S. B. J., & Sharkey, J. (2014). Developing an agenda for research about policies to improve access to healthy foods in rural communities: a concept mapping study. *BMC public health, 14*, 592. https://doi.org/https://dx.doi.org/10.1186/1471-2458-14-592

Johnson, S., Van Hoye, A., Donaldson, A., Lemonnier, F., Rostan, F., & Vuillemin, A. (2020). Building health-promoting sports clubs: a participative concept mapping approach. *Public Health, 188*, 8-17. https://doi.org/https://dx.doi.org/10.1016/j.puhe.2020.08.029

Joukes, E., Cornet, R., de Bruijne, M. C., & de Keizer, N. F. (2016). Eliciting end-user expectations to guide the implementation process of a new electronic health record: A case study using concept mapping. *International journal of medical informatics, 87*, 111-117. https://doi.org/https://dx.doi.org/10.1016/j.ijmedinf.2015.12.014

Kabukye, J. K., de Keizer, N., & Cornet, R. (2020). Elicitation and prioritization of requirements for electronic health records for oncology in low resource settings: A concept mapping study. *International journal of medical informatics, 135*, 104055. https://doi.org/https://dx.doi.org/10.1016/j.ijmedinf.2019.104055

Kadambi, M., Audet, C. T., & Knish, S. (2010). Counseling higher education students: Counselors' positive experiences. *Journal of College Student Psychotherapy, 24*(3), 213-232. https://doi.org/http://dx.doi.org/10.1080/87568225.2010.486296

Kadambi, M., & Truscott, D. (2006). Concept mapping professionals' perceptions of reward and motive in providing sex offender treatment. *Journal of Offender Rehabilitation, 42*(4), 37-58. https://doi.org/http://dx.doi.org/10.1300/J076v42n04_03

Kadambi, M. A., Truscott, D., & Adams, B. B. B. B. B. B. B. B. B. D. D. G. G. G. G. H. I. J. K. K. K. K. K. M. M. N. P. P. P. P. (2008). Traumatizing aspects of providing counselling in community agencies to survivors of sexual violence: A concept map. *Canadian Journal of Counselling, 42*(3), 192-208.

http://ovidsp.ovid.com/ovidweb.cgi?T=JS&PAGE=reference&D=psyc6&NEWS=N&AN=2008-11663-003

Kazmerski, T. M., Prushinskaya, O. V., Hill, K., Nelson, E., Leonard, J., Mogren, K., Pitts, S. A. B., Roboff, J., Uluer, A., Emans, S. J., Miller, E., & Sawicki, G. S. (2019). Sexual and Reproductive Health of Young Women With Cystic Fibrosis: A Concept Mapping Study. *Academic pediatrics, 19*(3), 307-314. https://doi.org/https://dx.doi.org/10.1016/j.acap.2018.08.011

Keita, A. D., Whittaker, S., Wynter, J., Kidanu, T. W., Chhay, C., Cardel, M., & Gans, K. M. (2016). Applying concept mapping methodology to identify the perceptions of risk and protective factors for childhood obesity among Southeast Asian refugees. *Journal of Health Care for the Poor and Underserved, 27*(4), 1909-1933. https://doi.org/http://dx.doi.org/10.1353/hpu.2016.0171

Keller, A. O. (2021). A Healthy Life for African American Women Caregivers: A Concept Mapping Study. *Women's health issues : official publication of the Jacobs Institute of Women's Health, 31*(2), 130-139. https://doi.org/https://dx.doi.org/10.1016/j.whi.2020.11.011

Kelly, C. M., Baker, E. A., Brownson, R. C., & Schootman, M. (2007). Translating research into practice: Using concept mapping to determine locally relevant intervention strategies to increase physical activity. *Evaluation and Program Planning, 30*(3), 282-293. https://doi.org/http://dx.doi.org/10.1016/j.evalprogplan.2007.05.007

Kerr, C. W., Donnelly, J. P., Wright, S. T., Luczkiewicz, D. L., McKenzie, K. J., Hang, P. C., & Kuszczak, S. M. (2013). Progression of delirium in advanced illness: A multivariate model of caregiver and clinician perspectives. *Journal of Palliative Medicine, 16*(7), 768-773. https://doi.org/http://dx.doi.org/10.1089/jpm.2012.0561

Kiran, T., Wells, D., Okrainec, K., Kennedy, C., Devotta, K., Mabaya, G., Phillips, L., Lang, A., & O'Campo, P. (2020). Patient and caregiver priorities in the transition from hospital to home: results from province-wide group concept mapping. *BMJ quality & safety, 29*(5), 390-400. https://doi.org/https://dx.doi.org/10.1136/bmjqs-2019-009993

Klemm, S., van Broeckhuysen-Kloth, S., van Vliet, S., Oosterhuis, L., & Geenen, R. (2018). Personalized treatment outcomes in patients with somatoform disorder: A concept mapping study. *Journal of Psychosomatic Research, 109*, 19-24. https://doi.org/http://dx.doi.org/10.1016/j.jpsychores.2018.03.009

Klokker, L., Osborne, R., Waehrens, E. E., Norgaard, O., Bandak, E., Bliddal, H., & Henriksen, M. (2015). The concept of physical limitations in knee osteoarthritis: As viewed by patients and health professionals. *Quality of Life Research: An International Journal*

of Quality of Life Aspects of Treatment, Care & Rehabilitation, 24(10), 2423-2432. https://doi.org/http://dx.doi.org/10.1007/s11136-015-0976-9

Kool, M. B., Geenen, R., Egberts, M. R., Wanders, H., & Van Loey, N. E. (2017). Patients' perspectives on quality of life after burn. *Burns : journal of the International Society for Burn Injuries, 43*(4), 747-756. https://doi.org/https://dx.doi.org/10.1016/j.burns.2016.11.016

Larizza, M. F., Zukerman, I., Bohnert, F., Busija, L., Bentley, S. A., Russell, R. A., & Rees, G. (2014). In-home monitoring of older adults with vision impairment: exploring patients', caregivers' and professionals' views. *Journal of the American Medical Informatics Association : JAMIA, 21*(1), 56-63. https://doi.org/https://dx.doi.org/10.1136/amiajnl-2012-001586

Lebel, A., Cantinotti, M., Pampalon, R., Thériault, M., Smith, L. A., & Hamelin, A. (2011). Concept mapping of diet and physical activity: Uncovering local stakeholders perception in the Quebec City region. *Social Science & Medicine, 72*(3), 439-445. https://doi.org/10.1016/j.socscimed.2010.09.013

Leyns, C. C., Couvreur, N., Willems, S., & Van Hecke, A. (2021). Needs and resources of people with type 2 diabetes in peri-urban Cochabamba, Bolivia: a people-centred perspective. *International journal for equity in health, 20*(1), 104. https://doi.org/https://dx.doi.org/10.1186/s12939-021-01442-1

Leyns, C. C., De Maeseneer, J., & Willems, S. (2018). Using concept mapping to identify policy options and interventions towards people-centred health care services: a multi stakeholders perspective. *International journal for equity in health, 17*(1), 177. https://doi.org/https://dx.doi.org/10.1186/s12939-018-0895-9

Liddle, B. J., Kunkel, M. A., Kick, S. L., & Hauenstein, A. L. (1998). The gay, lesbian, and bisexual psychology faculty experience: A concept map. *Teaching of Psychology, 25*(1), 19-25. https://doi.org/http://dx.doi.org/10.1207/s15328023top2501_6

Lloyd-Evans, B., Bond, G. R., Ruud, T., Ivanecka, A., Gray, R., Osborn, D., Nolan, F., Henderson, C., Mason, O., Goater, N., Kelly, K., Ambler, G., Morant, N., Onyett, S., Lamb, D., Fahmy, S., Brown, E., Paterson, B., Sweeney, A., Hindle, D., Fullarton, K., Frerichs, J., & Johnson, S. (2016). Development of a measure of model fidelity for mental health Crisis Resolution Teams. *BMC Psychiatry, 16*. http://ovidsp.ovid.com/ovidweb.cgi?T=JS&PAGE=reference&D=psyc13&NEWS=N&AN=2016-58989-001

Lloyd, M. H., Johnson, T., & Brook, J. (2014). Illuminating the black box from within: Stakeholder perspectives on family drug court best practices. *Journal of Social Work*

Practice in the Addictions, 14(4), 378-401. https://doi.org/http://dx.doi.org/10.1080/1533256X.2014.958492

Lobb, R., Pinto, A. D., & Lofters, A. (2013). Using concept mapping in the knowledge-to-action process to compare stakeholder opinions on barriers to use of cancer screening among South Asians. *Implementation science : IS, 8*, 37. https://doi.org/https://dx.doi.org/10.1186/1748-5908-8-37

Macdiarmid, J. I., Loe, J., Douglas, F., Ludbrook, A., Comerford, C., & McNeill, G. (2011). Developing a timeline for evaluating public health nutrition policy interventions. What are the outcomes and when should we expect to see them? *Public health nutrition, 14*(4), 729-739. https://doi.org/https://dx.doi.org/10.1017/S1368980010002168

Macleod, A., Busija, L., & McCabe, M. (2020). Mapping the Perceived Sexuality of Heterosexual Men and Women in Mid- and Later Life: A Mixed-Methods Study. *Sexual medicine, 8*(1), 84-99. https://doi.org/https://dx.doi.org/10.1016/j.esxm.2019.10.001

Mahabir, D. F., O'Campo, P., Lofters, A., Shankardass, K., Salmon, C., & Muntaner, C. (2021). Classism and Everyday Racism as Experienced by Racialized Health Care Users: A Concept Mapping Study. *International journal of health services : planning, administration, evaluation*, 207314211014782. https://doi.org/https://dx.doi.org/10.1177/00207314211014782

Mahabir, D. F., O'Campo, P., Lofters, A., Shankardass, K., Salmon, C., & Muntaner, C. (2021). Experiences of everyday racism in Toronto's health care system: a concept mapping study. *International journal for equity in health, 20*(1), 74. https://doi.org/https://dx.doi.org/10.1186/s12939-021-01410-9

Mâsse, L. C., O'Connor, T. M., Tu, A. W., Hughes, S. O., Beauchamp, M. R., & Baranowski, T. (2017). Conceptualizing physical activity parenting practices using expert informed concept mapping analysis. *BMC public health, 17*, 1-11. https://doi.org/10.1186/s12889-017-4487-1

McCaffrey, S. A., Chiauzzi, E., Chan, C., & Hoole, M. (2019). Understanding 'good health care' from the patient's perspective: Development of a conceptual model using group concept mapping. *The Patient: Patient-Centered Outcomes Research, 12*(1), 83-95. https://doi.org/http://dx.doi.org/10.1007/s40271-018-0320-x

McFall, S. L., Mullen, P. D., Byrd, T. L., Cantor, S. B., Le, Y.-C., Torres-Vigil, I., Pettaway, C., & Volk, R. J. (2015). Treatment decisions for localized prostate cancer: A concept mapping approach. *Health Expectations: An International Journal of Public*

Participation in Health Care & Health Policy, 18(6), 2079-2090. https://doi.org/http://dx.doi.org/10.1111/hex.12175

McFall, S. L., Ureda, J., Byrd, T. L., Valdes, A., Morales, P., Scott, D. B., Williams, D., Calderon-Mora, J., Casillas, M. E., & Chan, E. C. (2009). What is needed for informed decisions about prostate cancer screening: perspectives of African-American and Hispanic men. *Health Education Research, 24*(2), 280-291. https://doi.org/her/cyn018

McMahon, S., Burnham, J., & Banyard, V. L. (2020). Bystander intervention as a prevention strategy for campus sexual violence: Perceptions of historically minoritized college students. *Prevention Science*, No-Specified. https://doi.org/http://dx.doi.org/10.1007/s11121-020-01134-2

McNeish, R., Walker, C., Massey, O., & Tran, Q. (2020). Using concept mapping to operationalize mental well-being for men and boys. *American Journal of Community Psychology, 66*(1-2), 14-23. https://doi.org/http://dx.doi.org/10.1002/ajcp.12419

Mead, E. L., Johnson, S. L., Siddiqui, J., Butler, J., III, Kirchner, T., & Feldman, R. H. (2018). Beyond blunts: Reasons for cigarette and cigar use among African American young adult dual users. *Addiction Research & Theory, 26*(5), 349-360. https://doi.org/http://dx.doi.org/10.1080/16066359.2017.1366456

Melvin, K., Meyer, C., & Scarinci, N. (2020). What does a family who is "engaged" in early intervention look like? Perspectives of Australian speech-language pathologists. *International Journal of Speech-Language Pathology*, 1-11. https://doi.org/https://dx.doi.org/10.1080/17549507.2020.1784279

Miller, L. C., Rosas, S. R., & Hall, K. (2012). Using concept mapping to describe sources of information for public health and school nursing practice. *Journal of Research in Nursing, 17*(5), 466-481. https://doi.org/http://dx.doi.org/10.1177/1744987111403883

Mills, S. L., Bergeron, K., & Perez, G. (2015). Using concept mapping to develop a strategy for self-management support for underserved populations living with chronic conditions, British Columbia, August 2013-June 2014. *Preventing Chronic Disease: Public Health Research, Practice, and Policy, 12*. http://ovidsp.ovid.com/ovidweb.cgi?T=JS&PAGE=reference&D=psyc12&NEWS=N&AN=2016-25364-001

Moreno, M. A., Jelenchick, L. A., & Christakis, D. A. (2013). Problematic internet use among older adolescents: A conceptual framework. *Computers in Human Behavior, 29*(4), 1879-1887. https://doi.org/http://dx.doi.org/10.1016/j.chb.2013.01.053

Mpofu, E., Lawrence, F., Ngoma, M. S., Siziya, S., & Malungo, J. R. S. (2008). Mapping an HIV/STD prevention curriculum for Zambian in-school settings. *Special Issue: Culture and human development, 43*(2), 97-106. https://doi.org/http://dx.doi.org/10.1080/00207590701859267

Nabitz, U., van Den Brink, W., & Jansen, P. (2005). Using concept mapping to design an indicator framework for addiction treatment centres. *International journal for quality in health care : journal of the International Society for Quality in Health Care, 17*(3), 193-201. http://ovidsp.ovid.com/ovidweb.cgi?T=JS&PAGE=reference&D=med6&NEWS=N&AN=15831547

Nabitz, U., van den Brink, W., & Walburg, J. (2005). A quality framework for addiction treatment programs. *Addictive Behaviors, 30*(6), 1254-1260. https://doi.org/http://dx.doi.org/10.1016/j.addbeh.2004.12.006

Nalavany, B. A., Carawan, L. W., & Rennick, R. A. (2011). Psychosocial experiences associated with confirmed and self-identified dyslexia: a participant-driven concept map of adult perspectives. *Journal of Learning Disabilities, 44*(1), 63-79. https://doi.org/https://dx.doi.org/10.1177/0022219410374237

Nielsen, K. T., Klokker, L., Guidetti, S., & Waehrens, E. E. (2019). Identifying, organizing and prioritizing ideas on how to enhance ADL ability. *Scandinavian journal of occupational therapy, 26*(5), 382-393. https://doi.org/https://dx.doi.org/10.1080/11038128.2018.1424235

Nielsen, K. T., Rasmussen, M. U., Overgaard, A. F., Klokker, L., Christensen, R., & Waehrens, E. E. (2020). Identifying values and preferences around the choice of analgesia for patients with acute trauma pain in emergency and prehospital settings: using group concept mapping methodology. *BMJ open, 10*(3), e031863. https://doi.org/https://dx.doi.org/10.1136/bmjopen-2019-031863

Niemeijer, A., Frederiks, B., Depla, M., Eefsting, J., & Hertogh, C. (2013). The place of surveillance technology in residential care for people with intellectual disabilities: Is there an ideal model of application. *Journal of Intellectual Disability Research, 57*(3), 201-215. https://doi.org/http://dx.doi.org/10.1111/j.1365-2788.2011.01526.x

Niemeijer, A. R., Frederiks, B. J. M., Depla, M. F. I. A., Legemaate, J., Eefsting, J. A., & Hertogh, C. M. P. M. (2011). The ideal application of surveillance technology in residential care for people with dementia. *Journal of Medical Ethics: Journal of the Institute of Medical Ethics, 37*(5), 303-310. https://doi.org/http://dx.doi.org/10.1136/jme.2010.040774

Nijman, J. L., Sixma, H., van Triest, B., Keus, R. B., & Hendriks, M. (2012). The quality of radiation care: the results of focus group interviews and concept mapping to explore the patient's perspective. *Radiotherapy and oncology : journal of the European Society for Therapeutic Radiology and Oncology, 102*(1), 154-160. https://doi.org/https://dx.doi.org/10.1016/j.radonc.2011.08.005

Nijs, S., Taminiau, E. F., Frielink, N., & Embregts, P. J. C. M. (2019). Stakeholders' perspectives on how to improve the support for persons with an intellectual disability and challenging behaviors: A concept mapping study. *International Journal of Developmental Disabilities*, No-Specified. https://doi.org/http://dx.doi.org/10.1080/20473869.2019.1690859

Noel Racine, A., Garbarino, J. M., Corrion, K., D'Arripe-Longueville, F., Massiera, B., & Vuillemin, A. (2020). Perceptions of barriers and levers of health-enhancing physical activity policies in mid-size French municipalities. *Health research policy and systems, 18*(1), 62. https://doi.org/https://dx.doi.org/10.1186/s12961-020-00575-z

Nowicki, E. A., Brown, J., & Stepien, M. (2014). Children's structured conceptualizations of their beliefs on the causes of learning difficulties. *Journal of Mixed Methods Research, 8*(1), 69-82. https://doi.org/http://dx.doi.org/10.1177/1558689813490834

Noyes-Grosser, D. M., Rosas, S. R., Goldman, A., Elbaum, B., Romanczyk, R., & Callahan, E. H. (2013). Conceptualizing child and family outcomes of early intervention services for children with ASD and their families. *Journal of Early Intervention, 35*(4), 332-354. https://doi.org/http://dx.doi.org/10.1177/1053815114551415

Nuampa, S., Tilokskulchai, F., Patil, C. L., Sinsuksai, N., & Phahuwatanakorn, W. (2019). Factors related to exclusive breastfeeding in Thai adolescent mothers: Concept mapping approach. *Maternal & child nutrition, 15*(2), e12714. https://doi.org/https://dx.doi.org/10.1111/mcn.12714

Nureeva, L., Brunso, K., & Lahteenmaki, L. (2016). Exploring self-regulatory strategies for eating behaviour in Danish adolescents. *Young Consumers, 17*(2), 155-167. https://doi.org/http://dx.doi.org/10.1108/YC-10-2015-00565

O'Campo, P., Burke, J., Peak, G. L., McDonnell, K. A., & Gielen, A. C. (2005). Uncovering neighbourhood influences on intimate partner violence using concept mapping. *Journal of Epidemiology and Community Health, 59*(7), 603-608. https://doi.org/http://dx.doi.org/10.1136/jech.2004.027227

O'Campo, P., Salmon, C., & Burke, J. (2009). Neighbourhoods and mental well-being: what are the pathways? *Health & place, 15*(1), 56-68. https://doi.org/https://dx.doi.org/10.1016/j.healthplace.2008.02.004

O'Campo, P., Smylie, J., Minh, A., Omand, M., & Cyriac, A. (2015). Conceptualizing acts and behaviours that comprise intimate partner violence: A concept map. *Health Expectations: An International Journal of Public Participation in Health Care & Health Policy, 18*(6), 1968-1981. https://doi.org/http://dx.doi.org/10.1111/hex.12291

Ogden, K., Barr, J., & Greenfield, D. (2017). Determining requirements for patient-centred care: a participatory concept mapping study. *BMC health services research, 17*(1), 780. https://doi.org/https://dx.doi.org/10.1186/s12913-017-2741-y

Onken, S. J. (2018). Mental health consumer concept mapping of supportive community. *Evaluation and Program Planning, 71*, 36-45. https://doi.org/https://dx.doi.org/10.1016/j.evalprogplan.2018.08.001

Pammer, W., Haney, M., Lmhc, N., Wood, B. M., Brooks, R. G., Morse, K., Hicks, P., Handler, E. G., Rogers, H., & Jennett, P. (2001). Use of telehealth technology to extend child protection team services. *Pediatrics, 108*(3), 584-590. http://ovidsp.ovid.com/ovidweb.cgi?T=JS&PAGE=reference&D=med4&NEWS=N&AN=11533322

Parsons, J. A., Mamdani, M., Bhattacharyya, O., Fortin, C. M., Melo, M., Salmon, C., Raptis, S. R., Bain, D., & O'Campo, P. (2011). Narcotic analgesic utilization amongst injured workers: using concept mapping to understand current issues from the perspectives of physicians and pharmacists. *BMC health services research, 11*, 280. https://doi.org/https://dx.doi.org/10.1186/1472-6963-11-280

Paulson, B. L., & Everall, R. D. (2003). Suicidal Adolescents: Helpful Aspects of Psychotherapy. *Archives of Suicide Research, 7*(4), 309-321. https://doi.org/http://dx.doi.org/10.1080/713848939

Paulson, B. L., Everall, R. D., & Stuart, J. (2001). Client perceptions of hindering experiences in counselling. *Counselling & Psychotherapy Research, 1*(1), 53-61. http://ez.library.latrobe.edu.au/login?url=http://search.ebscohost.com/login.aspx?direct=true&db=cin20&AN=106838570&site=ehost-live&scope=site

Paulson, B. L., & Worth, M. (2002). Counseling for suicide: Client perspectives. *Journal of Counseling & Development, 80*(1), 86-93. https://doi.org/http://dx.doi.org/10.1002/j.1556-6678.2002.tb00170.x

Pauly, B., Martin, W., Perkin, K., van Roode, T., Kwan, A., Patterson, T., Tong, S., Prescott, C., Wallace, B., Hancock, T., & MacDonald, M. (2018). Critical considerations for the practical utility of health equity tools: a concept mapping study. *International journal for equity in health, 17*(1), 48. https://doi.org/https://dx.doi.org/10.1186/s12939-018-0764-6

Pearson, T., Chandler, R., McCreary, L. L., Patil, C. L., & McFarlin, B. L. (2020). Perceptions of African American Women and Health Care Professionals Related to Pre-Exposure Prophylaxis to Prevent HIV. *Journal of obstetric, gynecologic, and neonatal nursing : JOGNN, 49*(6), 571-580. https://doi.org/https://dx.doi.org/10.1016/j.jogn.2020.07.003

Phad, A., Johnston, S., Tabak, R. G., Mazzucca, S., & Haire-Joshu, D. (2019). Developing priorities to achieve health equity through diabetes translation research: a concept mapping study. *BMJ open diabetes research & care, 7*(1), e000851. https://doi.org/https://dx.doi.org/10.1136/bmjdrc-2019-000851

Piedra, L. M., Ridings, J., Howe, M. J. K., Smith, J. L., O'Brien, C., Howard, A., & Conrad, K. J. (2020). Stakeholders' Ideas About Positive Aging for Latinos: A Conceptual Map. *Journal of applied gerontology : the official journal of the Southern Gerontological Society*, 733464820935749. https://doi.org/https://dx.doi.org/10.1177/0733464820935749

Poost-Foroosh, L., Jennings, M. B., & Cheesman, M. F. (2015). Comparisons of client and clinician views of the importance of factors in client-clinician interaction in hearing aid purchase decisions. *Journal of the American Academy of Audiology, 26*(3), 247-259. https://doi.org/https://dx.doi.org/10.3766/jaaa.26.3.5

Powell, B. J., Stanick, C. F., Halko, H. M., Dorsey, C. N., Weiner, B. J., Barwick, M. A., Damschroder, L. J., Wensing, M., Wolfenden, L., & Lewis, C. C. (2017). Toward criteria for pragmatic measurement in implementation research and practice: a stakeholder-driven approach using concept mapping. *Implementation science : IS, 12*(1), 118. https://doi.org/https://dx.doi.org/10.1186/s13012-017-0649-x

Rachlis, B., Ahmad, F., van Lettow, M., Muula, A. S., Semba, M., & Cole, D. C. (2013). Using concept mapping to explore why patients become lost to follow up from an antiretroviral therapy program in the Zomba District of Malawi. *BMC health services research, 13*, 210. https://doi.org/https://dx.doi.org/10.1186/1472-6963-13-210

Rachlis, B., Nam, S., Rosenes, R., Santoni, T., Peck, R., Betts, A., Kendall, C., Yoong, D., Sharp, A., Gauvin, H., Goddard, L., Owino, M., Rourke, S. B., & Antoniou, T. (2021). Using concept mapping to explore the challenges associated with affording and accessing

medications among people living with HIV in Ontario, Canada. *AIDS Care, 33*(6), 827-832. https://doi.org/10.1080/09540121.2020.1770182

Rainey, L., van der Waal, D., Donnelly, L. S., Evans, D. G., Wengstrom, Y., & Broeders, M. (2018). Women's decision-making regarding riskstratified breast cancer screening and prevention from the perspective of international healthcare professionals. *PLoS ONE, 13*(6). https://doi.org/http://dx.doi.org/10.1371/journal.pone.0197772

Ridde, V. (2008). Equity and health policy in Africa: using concept mapping in Moore (Burkina Faso). *BMC health services research, 8*, 90. https://doi.org/https://dx.doi.org/10.1186/1472-6963-8-90

Rising, K. L., Doyle, S. K., Powell, R. E., Doty, A. M. B., LaNoue, M., & Gerolamo, A. M. (2019). Use of Group Concept Mapping to Identify Patient Domains of Uncertainty That Contribute to Emergency Department Use. *Journal of emergency nursing, 45*(1), 46-53. https://doi.org/https://dx.doi.org/10.1016/j.jen.2018.05.015

Robinson, J. M., & Trochim, W. M. K. (2007). An examination of community members', researchers' and health professionals' perceptions of barriers to minority participation in medical research: an application of concept mapping. *Ethnicity & Health, 12*(5), 521-539. http://ovidsp.ovid.com/ovidweb.cgi?T=JS&PAGE=reference&D=med6&NEWS=N&AN=17978947

Robinson, L. J., Stephens, N. M., Wilson, S., Graham, L., & Hackett, K. L. (2020). Conceptualizing the key components of rehabilitation following major musculoskeletal trauma: A mixed methods service evaluation. *Journal of evaluation in clinical practice, 26*(5), 1436-1447. https://doi.org/https://dx.doi.org/10.1111/jep.13331

Roeg, D., van de Goor, I., & Garretsen, H. (2005). Towards quality indicators for assertive outreach programmes for severely impaired substance abusers: concept mapping with Dutch experts. *International journal for quality in health care : journal of the International Society for Quality in Health Care, 17*(3), 203-208. http://ovidsp.ovid.com/ovidweb.cgi?T=JS&PAGE=reference&D=med6&NEWS=N&AN=15788464

Roodenrijs, N. M. T., van der Goes, M. C., Welsing, P. M. J., van Oorschot, E. P. C., Nikiphorou, E., Nijhof, N. C., Tekstra, J., Lafeber, F. P. J. G., Jacobs, J. W. G., van Laar, J. M., & Geenen, R. (2021). Non-adherence in difficult-to-treat rheumatoid arthritis from the perspectives of patients and rheumatologists: a concept mapping study. *Rheumatology (Oxford, England)*. https://doi.org/https://dx.doi.org/10.1093/rheumatology/keab130

Rosenkotter, N., Achterberg, P. W., van Bon-Martens, M. J. H., Michelsen, K., van Oers, H. A. M., & Brand, H. (2016). Key features of an EU health information system: a concept mapping study. *European Journal of Public Health, 26*(1), 65-70. https://doi.org/https://dx.doi.org/10.1093/eurpub/ckv075

Rostad-Tollefsen, H. K., Kolset, S. O., Retterstol, K., Hesselberg, H., & Nordstrom, M. (2021). Factors influencing the opportunities of supporting staff to promote a healthy diet in adults with intellectual disabilities. *Journal of applied research in intellectual disabilities : JARID, 34*(3), 733-741. https://doi.org/https://dx.doi.org/10.1111/jar.12846

Rouhi, M., Stirling, C. M., & Crisp, E. P. (2019). Mothers' views of health problems in the 12 months after childbirth: A concept mapping study. *Journal of Advanced Nursing, 75*(12), 3702-3714. https://doi.org/https://dx.doi.org/10.1111/jan.14187

Ruud, M. P., Raanaas, R. K., & Bjelland, M. (2016). Caregivers' perception of factors associated with a healthy diet among people with intellectual disability living in community residences: A Concept mapping method. *Research in Developmental Disabilities, 59*, 202-210. https://doi.org/http://dx.doi.org/10.1016/j.ridd.2016.09.006

Ryan, S. D., Nelson, N., & Siebert, C. F. (2009). Examining the facilitators and barriers faced by adoptive professionals delivering post-placement services. *Children and Youth Services Review, 31*(5), 584-593. https://doi.org/http://dx.doi.org/10.1016/j.childyouth.2008.11.003

Schell, S. F., Luke, D. A., Schooley, M. W., Elliott, M. B., Herbers, S. H., Mueller, N. B., & Bunger, A. C. (2013). Public health program capacity for sustainability: a new framework. *Implementation science : IS, 8*, 15. https://doi.org/https://dx.doi.org/10.1186/1748-5908-8-15

Selten, E. M. H., Geenen, R., van der Laan, W. H., van der Meulen-Dilling, R. G., Schers, H. J., Nijhof, M. W., van den Ende, C. H. M., & Vriezekolk, J. E. (2017). Hierarchical structure and importance of patients' reasons for treatment choices in knee and hip osteoarthritis: a concept mapping study. *Rheumatology (Oxford, England), 56*(2), 271-278. https://doi.org/https://dx.doi.org/10.1093/rheumatology/kew409

Sheppard, A. J., Salmon, C., Balasubramaniam, P., Parsons, J., Singh, G., Jabbar, A., Zaidi, Q., Scott, A., Nisenbaum, R., Dunn, J., Ramsay, J., Haque, N., & O'Campo, P. (2012). Are residents of downtown Toronto influenced by their urban neighbourhoods? Using concept mapping to examine neighbourhood characteristics and their perceived impact on self-rated mental well-being. *International journal of health geographics,*

11, 31. http://ovidsp.ovid.com/ovidweb.cgi?T=JS&PAGE=reference&D=med9&NEWS=N&AN=22862839

Shorkey, C., Windsor, L. C., & Spence, R. (2009). Systematic assessment of culturally competent chemical dependence treatment services for African Americans. *Journal of Ethnicity in Substance Abuse, 8*(2), 113-128. https://doi.org/https://dx.doi.org/10.1080/15332640902896943

Simpson, A. J., & Bedi, R. P. (2012). The therapeutic alliance: Clients' categorization of client-identified factors. *Canadian Journal of Counselling and Psychotherapy, 46*(4), 344-366. http://ovidsp.ovid.com/ovidweb.cgi?T=JS&PAGE=reference&D=psyc9&NEWS=N&AN=2013-26512-006

Singer, B. A., Keith, S., Howerter, A., Doll, H., Pham, T., & Mehta, R. (2021). A Study Comparing Patient and Clinician Perspectives of Treatments for Multiple Sclerosis via Group Concept Mapping. *Patient preference and adherence, 15*, 975-987. https://doi.org/https://dx.doi.org/10.2147/PPA.S297052

Skempes, D., Melvin, J., von Groote, P., Stucki, G., & Bickenbach, J. (2018). Using concept mapping to develop a human rights based indicator framework to assess country efforts to strengthen rehabilitation provision and policy: the Rehabilitation System Diagnosis and Dialogue framework (RESYST). *Globalization and health, 14*(1), 96. https://doi.org/https://dx.doi.org/10.1186/s12992-018-0410-5

Smith, F., Alexandersson, P., Bergman, B., Vaughn, L., & Hellstrom, A. (2019). Fourteen years of quality improvement education in healthcare: a utilisation-focused evaluation using concept mapping. *BMJ open quality, 8*(4), e000795. https://doi.org/https://dx.doi.org/10.1136/bmjoq-2019-000795

Smith, F., Gunnarsdottir, K. A., Genell, A., McLinden, D., Vaughn, L., Garelius, H., Nilsson-Ehle, H., Lonqvist, U., & Bjork-Eriksson, T. (2019). Evaluating the implementation and use of the regional cancer plan in Western Sweden through concept mapping. *International journal for quality in health care : journal of the International Society for Quality in Health Care, 31*(7), 44-52. https://doi.org/https://dx.doi.org/10.1093/intqhc/mzy241

Smith, S., D'Cruz, G., Gray, R., Flaherty, H., Ivanecka, A., & Deane, K. H. O. (2015). A Concept Map of What Helps People with HD Live with their Condition. *Journal of Huntington's disease, 4*(3), 261-270. https://doi.org/https://dx.doi.org/10.3233/JHD-150161

Snider, C. E., Kirst, M., Abubakar, S., Ahmad, F., & Nathens, A. B. (2010). Community-based participatory research: development of an emergency department-based youth violence intervention using concept mapping. *Academic emergency medicine : official journal of the Society for Academic Emergency Medicine, 17*(8), 877-885. https://doi.org/https://dx.doi.org/10.1111/j.1553-2712.2010.00810.x

Soellner, R., Lenartz, N., & Rudinger, G. (2017). Concept mapping as an approach for expert-guided model building: The example of health literacy. *Evaluation and Program Planning, 60*, 245-253. https://doi.org/http://dx.doi.org/10.1016/j.evalprogplan.2016.10.007

Sommerfeld, D. H., Jaramillo, E. T., Lujan, E., Haozous, E., & Willging, C. E. (2021). Health Care Access and Utilization for American Indian Elders: A Concept-Mapping Study. *The journals of gerontology. Series B, Psychological sciences and social sciences, 76*(1), 141-151. https://doi.org/https://dx.doi.org/10.1093/geronb/gbz112

Soule, E. K., Lee, J. G. L., Egan, K. L., Bode, K. M., Desrosiers, A. C., Guy, M. C., Breland, A., & Fagan, P. (2020). "I cannot live without my vape": Electronic cigarette user-identified indicators of vaping dependence. *Drug and Alcohol Dependence, 209*. https://doi.org/http://dx.doi.org/10.1016/j.drugalcdep.2020.107886

Soule, E. K., Lopez, A. A., Guy, M. C., & Cobb, C. O. (2016). Reasons for using flavored liquids among electronic cigarette users: A concept mapping study. *Drug and Alcohol Dependence, 166*, 168-176. https://doi.org/http://dx.doi.org/10.1016/j.drugalcdep.2016.07.007

Soule, E. K., Maloney, S. F., Eissenberg, T., Guy, M. C., & Fagan, P. (2018). User-identified electronic cigarette behavioral strategies and device characteristics for cigarette smoking reduction. *Addictive Behaviors, 79*, 93-101. https://doi.org/10.1016/j.addbeh.2017.12.010

Soule, E. K., Maloney, S. F., Guy, M. C., Eissenberg, T., & Fagan, P. (2017). User identified positive outcome expectancies of electronic cigarette use: A concept mapping study. *Psychology of Addictive Behaviors, 31*(3), 343-353. https://doi.org/http://dx.doi.org/10.1037/adb0000263

Soule, E. K., Mayne, S., Snipes, W., Guy, M. C., Breland, A., & Fagan, P. (2020). Impacts of COVID-19 on Electronic Cigarette Purchasing, Use and Related Behaviors. *International journal of environmental research and public health, 17*(18). https://doi.org/https://dx.doi.org/10.3390/ijerph17186762

Soule, E. K., Nasim, A., & Rosas, S. (2016). Adverse effects of electronic cigarette use: A concept mapping approach. *Nicotine & Tobacco Research, 18*(5), 678-685. https://doi.org/http://dx.doi.org/10.1093/ntr/ntv246

Soule, E. K., Rosas, S. R., & Nasim, A. (2016). Reasons for electronic cigarette use beyond cigarette smoking cessation: A concept mapping approach. *Addictive Behaviors, 56*, 41-50. https://doi.org/https://dx.doi.org/10.1016/j.addbeh.2016.01.008

Staley, K., Donaldson, A., Randle, E., Nicholson, M., O'Halloran, P., Nelson, R., & Cameron, M. (2019). Challenges for sport organisations developing and delivering non-traditional social sport products for insufficiently active populations. *Australian and New Zealand journal of public health, 43*(4), 373-381. https://doi.org/https://dx.doi.org/10.1111/1753-6405.12912

Stillman, F., Hoang, M., Linton, R., Ritthiphakdee, B., & Trochim, W. (2008). Mapping tobacco industry strategies in South East Asia for action planning and surveillance. *Tobacco control, 17*(1), e1. https://doi.org/https://dx.doi.org/10.1136/tc.2006.017988

Stillman, F. A., Schmitt, C. L., & Rosas, S. R. (2012). Opportunity for collaboration: a conceptual model of success in tobacco control and cancer prevention. *Preventing chronic disease, 9*, E02. http://ovidsp.ovid.com/ovidweb.cgi?T=JS&PAGE=reference&D=med9&NEWS=N&AN=22172169

Stolk-Vos, A. C., van de Klundert, J. J., Maijers, N., Zijlmans, B. L. M., & Busschbach, J. J. V. (2017). Multi-stakeholder perspectives in defining health-services quality in cataract care. *International journal for quality in health care : journal of the International Society for Quality in Health Care, 29*(4), 470-476. https://doi.org/https://dx.doi.org/10.1093/intqhc/mzx048

Stoyanov, S., Boshuizen, H., Groene, O., van der Klink, M., Kicken, W., Drachsler, H., & Barach, P. (2012). Mapping and assessing clinical handover training interventions. *BMJ quality & safety, 21 Suppl 1*, i50-57. https://doi.org/https://dx.doi.org/10.1136/bmjqs-2012-001169

Strassheim, V., Deary, V., Webster, D. A., Douglas, J., Newton, J. L., & Hackett, K. L. (2021). Conceptualizing the benefits of a group exercise program developed for those with chronic fatigue: a mixed methods clinical evaluation. *Disability and rehabilitation, 43*(5), 657-667. https://doi.org/https://dx.doi.org/10.1080/09638288.2019.1636315

Surko, M., Pasti, L. W., Whitlock, J., & Benson, D. A. (2006). Selecting statewide youth development outcome indicators. *Journal of public health management and practice*

: *JPHMP, Suppl*, S72-78. http://ovidsp.ovid.com/ovidweb.cgi?T=JS&PAGE=reference&D=med6&NEWS=N&AN=17035907

Svobodova, I., Filakovska Bobakova, D., Bosakova, L., & Dankulincova Veselska, Z. (2021). How to improve access to health care for Roma living in social exclusion: a concept mapping study. *International journal for equity in health, 20*(1), 61. https://doi.org/https://dx.doi.org/10.1186/s12939-021-01396-4

Sweegers, M. G., Buffart, L. M., van Veldhuizen, W. M., Geleijn, E., Verheul, H. M. W., Brug, J., Chinapaw, M. J. M., & Altenburg, T. M. (2019). How Does a Supervised Exercise Program Improve Quality of Life in Patients with Cancer? A Concept Mapping Study Examining Patients' Perspectives. *The oncologist, 24*(6), e374-e383. https://doi.org/https://dx.doi.org/10.1634/theoncologist.2017-0613

Szaflarski, M., Vaughn, L. M., McLinden, D., Wess, Y., & Ruffner, A. (2015). Using concept mapping to mobilize a Black faith community to address HIV. *International public health journal, 7*(1), 117-130. http://ovidsp.ovid.com/ovidweb.cgi?T=JS&PAGE=reference&D=pmnm3&NEWS=N&AN=28239439

Tabak, R. G., Padek, M. M., Kerner, J. F., Stange, K. C., Proctor, E. K., Dobbins, M. J., Colditz, G. A., Chambers, D. A., & Brownson, R. C. (2017). Dissemination and implementation science training needs: Insights from practitioners and researchers. *Special Issue: Prevention Research Centers Program's 30th anniversary celebration, 52*(3, Suppl 3), S322-S329. https://doi.org/http://dx.doi.org/10.1016/j.amepre.2016.10.005

Thepha, T., Marais, D., Bell, J., & Muangpin, S. (2019). Concept mapping to reach consensus on a 6-month exclusive breastfeeding strategy model to improve the rate in Northeast Thailand. *Maternal & child nutrition, 15*(4), e12823. https://doi.org/https://dx.doi.org/10.1111/mcn.12823

Trezona, A., Dodson, S., & Osborne, R. H. (2017). Development of the organisational health literacy responsiveness (Org-HLR) framework in collaboration with health and social services professionals. *BMC health services research, 17*(1), 513. https://doi.org/https://dx.doi.org/10.1186/s12913-017-2465-z

Trochim, W. M., Cabrera, D. A., Milstein, B., Gallagher, R. S., & Leischow, S. J. (2006). Practical challenges of systems thinking and modeling in public health. *American Journal of Public Health, 96*(3), 538-546. http://ovidsp.ovid.com/ovidweb.cgi?T=JS&PAGE=reference&D=med6&NEWS=N&AN=16449581

Trochim, W. M., Cook, J. A., & Setze, R. J. (1994). Using concept mapping to develop a conceptual framework of staff's views of a supported employment program for individuals with severe mental illness. *Journal of Consulting and Clinical Psychology, 62*(4), 766-775. http://ovidsp.ovid.com/ovidweb.cgi?T=JS&PAGE=reference&D=med3&NEWS=N&AN=7962880

Trochim, W. M. K., Milstein, B., Wood, B. J., Jackson, S., & Pressler, V. (2004). Setting objectives for community and systems change: an application of concept mapping for planning a statewide health improvement initiative. *Health Promotion Practice, 5*(1), 8-10. http://ovidsp.ovid.com/ovidweb.cgi?T=JS&PAGE=reference&D=med5&NEWS=N&AN=14965431

Trudeau, K. J., Ainscough, J. L., Pujol, L. A., & Charity, S. (2010). What arthritis pain practitioners and patients want in an online self-management programme. *Musculoskeletal care, 8*(4), 189-196. https://doi.org/https://dx.doi.org/10.1002/msc.183

Tubbing, L., Harting, J., & Stronks, K. (2015). Unravelling the concept of integrated public health policy: Concept mapping with Dutch experts from science, policy, and practice. *Health Policy, 119*(6), 749-759. https://doi.org/http://dx.doi.org/10.1016/j.healthpol.2014.12.020

Urbanoski, K., Pauly, B., Inglis, D., Cameron, F., Haddad, T., Phillips, J., Phillips, P., Rosen, C., Schlotter, G., Hartney, E., & Wallace, B. (2020). Defining culturally safe primary care for people who use substances: a participatory concept mapping study. *BMC health services research, 20*(1), 1060. https://doi.org/https://dx.doi.org/10.1186/s12913-020-05915-x

van Bon-Martens, M. J. H., Achterberg, P. W., van de Goor, I. A. M., & van Oers, H. A. M. (2012). Towards quality criteria for regional public health reporting: concept mapping with Dutch experts. *European Journal of Public Health, 22*(3), 337-342. https://doi.org/http://dx.doi.org/10.1093/eurpub/ckr016

van den Dungen, C., Hoeymans, N., Schellevis, F. G., & van Oers, H. J. A. M. (2013). Quality aspects of Dutch general practice-based data: A conceptual approach. *Family Practice, 30*(3), 355-361. https://doi.org/http://dx.doi.org/10.1093/fampra/cms082

van Engen-Verheul, M. M., Peek, N., Haafkens, J. A., Joukes, E., Vromen, T., Jaspers, M. W. M., & de Keizer, N. F. (2017). What is needed to implement a web-based audit and feedback intervention with outreach visits to improve care quality: A concept mapping study among cardiac rehabilitation teams. *International journal of medical*

informatics, 97, 76-85. https://doi.org/https://dx.doi.org/10.1016/j.ijmedinf.2016.10.003

van Grieken, R. A., Verburg, H. F., Koeter, M. W. J., Stricker, J., Nabitz, U. W., & Schene, A. H. (2016). Helpful Factors in the Treatment of Depression from the Patient's, Carer's and Professional's Perspective: A Concept Map Study. *PLoS ONE, 11*(12), e0167719. https://doi.org/https://dx.doi.org/10.1371/journal.pone.0167719

van Krugten, F. C. W., Goorden, M., van Balkom, A. J. L. M., Spijker, J., Brouwer, W. B. F., & Hakkaart-van Roijen, L. (2018). Indicators to facilitate the early identification of patients with major depressive disorder in need of highly specialized care: A concept mapping study. *Depression and Anxiety, 35*(4), 346-352. https://doi.org/http://dx.doi.org/10.1002/da.22741

van Randeraad-van der Zee, C., Beurskens, A., Swinkels, R., Pool, J., Batterham, R., Osborne, R., de Vet, H., van Randeraad-van der Zee, C. H., Beurskens, A. J. H. M., Swinkels, R. A. H. M., Pool, J. J. M., Batterham, R. W., Osborne, R. H., & de Vet, H. C. W. (2016). The burden of neck pain: its meaning for persons with neck pain and healthcare providers, explored by concept mapping. *Quality of Life Research, 25*(5), 1219-1225. https://doi.org/10.1007/s11136-015-1149-6

Varekamp, I., Haafkens, J. A., Detaille, S. I., Tak, P. P., & van Dijk, F. J. H. (2005). Preventing work disability among employees with rheumatoid arthritis: what medical professionals can learn from the patients' perspective. *Arthritis and rheumatism, 53*(6), 965-972. http://ovidsp.ovid.com/ovidweb.cgi?T=JS&PAGE=reference&D=med6&NEWS=N&AN=16342108

Vaughn, L., Jacquez, F., Marschner, D., McLinden, D., & Vaughn, L. M. (2016). See what we say: using concept mapping to visualize Latino immigrant's strategies for health interventions. *International Journal of Public Health (Springer Nature), 61*(7), 837-845. https://doi.org/10.1007/s00038-016-0838-4

Vaughn, L. M., Jacquez, F., & McLinden, D. (2013). The use of concept mapping to identify community-driven intervention strategies for physical and mental health. *Health Promotion Practice, 14*(5), 675-685. https://doi.org/https://dx.doi.org/10.1177/1524839912462871

Vaughn, L. M., Sunny, C. E., Lindquist-Grantz, R., King, C., Brent, D., Boyd, S., & Grupp-Phelan, J. (2020). Successful Suicide Screening in the Pediatric Emergency Department: Youth, Parent, Researcher, and Clinician Perspectives. *Archives of suicide research : official journal of the International Academy for Suicide Research,*

24(sup1), 124-141. https://doi.org/https://dx.doi.org/10.1080/13811118.2018.1541034

Velonis, A., & Forst, L. (2018). Outreach to Low-Wage and Precarious Workers: Concept Mapping for Public Health Officers. *Journal of occupational and environmental medicine, 60*(11), e610-e617. https://doi.org/https://dx.doi.org/10.1097/JOM.0000000000001462

Velonis, A. J., Hebert-Beirne, J., Conroy, L. M., Hernandez, M., Castaneda, D., & Forst, L. (2020). Impact of precarious work on neighborhood health: Concept mapping by a community/academic partnership. *American journal of industrial medicine, 63*(1), 23-35. https://doi.org/https://dx.doi.org/10.1002/ajim.23055

Vinson, C. A. (2014). Using concept mapping to develop a conceptual framework for creating virtual communities of practice to translate cancer research into practice. *Preventing chronic disease, 11*, E68. https://doi.org/https://dx.doi.org/10.5888/pcd11.130280

Visek, A. J., Blake, E. F., Otterbein, M., Chandran, A., & Sylvetsky, A. C. (2019). SWEET MAPS: A Conceptualization of Low-Calorie Sweetener Consumption Among Young Adults. *Current developments in nutrition, 3*(4), nzy103. https://doi.org/https://dx.doi.org/10.1093/cdn/nzy103

Vishwanath, A., & Scamurra, S. D. (2007). Barriers to the adoption of electronic health records: using concept mapping to develop a comprehensive empirical model. *Health informatics journal, 13*(2), 119-134. http://ovidsp.ovid.com/ovidweb.cgi?T=JS&PAGE=reference&D=med6&NEWS=N&AN=17510224

Vives-Cases, C., Goicolea, I., Hernandez, A., Sanz-Barbero, B., Davo-Blanes, M., & La Parra-Casado, D. (2017). Priorities and strategies for improving Roma women's access to primary health care services in cases on intimate partner violence: a concept mapping study. *International journal for equity in health, 16*(1), 96. https://doi.org/https://dx.doi.org/10.1186/s12939-017-0594-y

Walker, D. C., Heiss, S., Donahue, J. M., & Brooks, J. M. (2020). Practitioners' perspectives on ethical issues within the treatment of eating disorders: Results from a concept mapping study. *International Journal of Eating Disorders, 53*(12), 1941-1951. https://doi.org/http://dx.doi.org/10.1002/eat.23381

Walker, R. E., Block, J., & Kawachi, I. (2012). Do residents of food deserts express different food buying preferences compared to residents of food oases? A mixed-methods analysis. *The International Journal of Behavioral Nutrition and Physical Activity, 9*. https://doi.org/http://dx.doi.org/10.1186/1479-5868-9-41

Wallace, C., Dale, F., Jones, G., O'Kane, J., Thomas, M., Wilson, L., & Pontin, D. (2018). Developing the health visitor concept of family resilience in Wales using Group Concept Mapping. *Rural and remote health, 18*(4), 4604. https://doi.org/https://dx.doi.org/10.22605/RRH4604

Wallace, L. S., Wexler, R. K., Miser, W. F., McDougle, L., & Haddox, J. D. (2013). Development and validation of the Patient Opioid Education Measure. *Journal of pain research, 6*, 663-681. https://doi.org/https://dx.doi.org/10.2147/JPR.S50715

Washington-Nortey, P.-M., & Serpell, Z. (2021). Parental expectations for children with intellectual disability or autism in Ghana and Zambia: A concept mapping study. *Research in Developmental Disabilities, 114*, 103989. https://doi.org/https://dx.doi.org/10.1016/j.ridd.2021.103989

Wee, S., Todd, M.-J., Oshiro, M., Greene, E., & Frye, V. (2016). Modifiers of Neighbors' Bystander Intervention in Intimate Partner Violence: A Concept Mapping Study. *Violence and gender, 3*(1), 55-63. http://ovidsp.ovid.com/ovidweb.cgi?T=JS&PAGE=reference&D=pmnm3&NEWS=N&AN=27626038

Wentink, C., Huijbers, M. J., Lucassen, P. L., van der Gouw, A., Kramers, C., Spijker, J., & Speckens, A. E. (2019). Enhancing shared decision making about discontinuation of antidepressant medication: a concept-mapping study in primary and secondary mental health care. *The British journal of general practice : the journal of the Royal College of General Practitioners, 69*(688), e777-e785. https://doi.org/https://dx.doi.org/10.3399/bjgp19X706001

Westergren, A., Edfors, E., Norberg, E., Stubbendorff, A., Hedin, G., Wetterstrand, M., Rosas, S. R., & Hagell, P. (2018). Computer-Based Training in Eating and Nutrition Facilitates Person-Centered Hospital Care: A Group Concept Mapping Study. *Computers, informatics, nursing : CIN, 36*(4), 199-207. https://doi.org/https://dx.doi.org/10.1097/CIN.0000000000000416

Wilberforce, M., Batten, E., Challis, D., Davies, L., Kelly, M. P., & Roberts, C. (2018). The patient experience in community mental health services for older people: a concept mapping approach to support the development of a new quality measure. *BMC health services research, 18*(1), 461. https://doi.org/https://dx.doi.org/10.1186/s12913-018-3231-6

Williams, M. T., Donnelly, J. P., Holmlund, T., & Battaglia, M. (2008). ALS: Family caregiver needs and quality of life. *Amyotrophic lateral sclerosis : official publication of the*

World Federation of Neurology Research Group on Motor Neuron Diseases, 9(5), 279-286. https://doi.org/https://dx.doi.org/10.1080/17482960801934148

Windsor, L. C., & Murugan, V. (2012). From the individual to the community: Perspectives about substance abuse services. *Journal of Social Work Practice in the Addictions, 12*(4), 412-433. https://doi.org/http://dx.doi.org/10.1080/1533256X.2012.728115

Winseman, J., Malik, A., Morison, J., & Balkoski, V. (2009). Students' views on factors affecting empathy in medical education. *Academic Psychiatry, 33*(6), 484-491. https://doi.org/http://dx.doi.org/10.1176/appi.ap.33.6.484

Wutzke, S., Roberts, N., Willis, C., Best, A., Wilson, A., & Trochim, W. (2017). Setting strategy for system change: using concept mapping to prioritise national action for chronic disease prevention. *Health research policy and systems, 15*(1), 69. https://doi.org/https://dx.doi.org/10.1186/s12961-017-0231-7
