# Supplementary material for: A Systematic Review to Inform the Development of a Reporting Guideline for Concept Mapping Research
Source: Methods Protoc. 2023 Oct 17;6(5):101. doi: 10.3390/mps6050101 (PMC10609252; doi:10.3390/mps6050101)
Supplement: Supplementary file 1 [file mps-06-00101-s001.zip › Supplementary document 6_List of 88 studies.pdf]

List of 88 included studies after post-hoc amendment

Aarons, G. A., Reeder, K., Sam-Agudu, N. A., Vorkoper, S., & Sturke, R. (2021). Implementation determinants and mechanisms for the prevention and treatment of adolescent HIV in sub-Saharan Africa: concept mapping of the NIH Fogarty International Center Adolescent HIV Implementation Science Alliance (AHISA) initiative. *Implementation science communications, 2*(1), 53. https://doi.org/https://dx.doi.org/10.1186/s43058-021-00156-3

Ageberg, E., Bunke, S., Lucander, K., Nilsen, P., & Donaldson, A. (2019). Facilitators to support the implementation of injury prevention training in youth handball: A concept mapping approach. *Scandinavian journal of medicine & science in sports, 29*(2), 275-285. https://doi.org/https://dx.doi.org/10.1111/sms.13323

Antoniou, T., Mishra, S., Matheson, F., Smith-Merrill, D., Challacombe, L., Rowe, J., DiCenso, A. M., Kouyoumdjian, F. G., Wobeser, W., Kendall, C., Loutfy, M., Tsang, J., Kanee, L., & Strike, C. (2019). Using concept mapping to inform the development of a transitional reintegration intervention program for formerly incarcerated people with HIV. *BMC health services research, 19*(1), 761. https://doi.org/https://dx.doi.org/10.1186/s12913-019-4595-y

Ast, R. S., Banyard, V. L., Burnham, J., & Edwards, K. M. (2021). Community conversations on relationship violence: Town variations in prevention perceptions through concept mapping. *American Journal of Community Psychology*, No-Specified. https://doi.org/http://dx.doi.org/10.1002/ajcp.12488

Belmon, L. S., Busch, V., van Stralen, M. M., Stijnman, D. P. M., Hidding, L. M., Harmsen, I. A., & Chinapaw, M. J. M. (2020). Child and Parent Perceived Determinants of Children's Inadequate Sleep Health. A Concept Mapping Study. *International journal of environmental research and public health, 17*(5). https://doi.org/https://dx.doi.org/10.3390/ijerph17051583

Bennett, R. J., Barr, C., Montano, J., Eikelboom, R. H., Saunders, G. H., Pronk, M., Preminger, J. E., Ferguson, M., Weinstein, B., Heffernan, E., van Leeuwen, L., Hickson, L., Timmer, B. H. B., Singh, G., Gerace, D., Cortis, A., & Bellekom, S. R. (2021). Identifying the approaches used by audiologists to address the psychosocial needs of their adult clients. *International Journal of Audiology, 60*(2), 104-114. https://doi.org/https://dx.doi.org/10.1080/14992027.2020.1817995

Bennett, R. J., Fletcher, S., Conway, N., & Barr, C. (2020). The role of the general practitioner in managing age-related hearing loss: perspectives of general practitioners, patients and practice staff. *BMC family practice, 21*(1), 87. https://doi.org/https://dx.doi.org/10.1186/s12875-020-01157-2

Bennett, R. J., Meyer, C. J., & Eikelboom, R. H. (2019). How do hearing aid owners acquire hearing aid management skills? *Journal of the American Academy of Audiology, 30*(6), 516-532. http://ovidsp.ovid.com/ovidweb.cgi?T=JS&PAGE=reference&D=psyc17&NEWS=N&AN=2019-46922-007

Brons, A., Braam, K., Timmerman, A., Broekema, A., Visser, B., van Ewijk, B., Terheggen-Lagro, S., Rutjes, N., van Leersum, H., Engelbert, R., Krose, B., Chinapaw, M., & Altenburg, T. (2019). Promoting Factors for Physical Activity in Children with Asthma Explored through Concept Mapping. *International journal of environmental research and public health, 16*(22). https://doi.org/https://dx.doi.org/10.3390/ijerph16224467

Brown, E., Topping, A., & Cheston, R. (2019). What are the barriers to accessing psychological therapy in Qatar: A concept mapping study. *Counselling & Psychotherapy Research, 19*(4), 441-454. https://doi.org/10.1002/capr.12252

Brown, J., Kapasi, A., Nowicki, E., & Cleversey, K. (2019). Expectations of youth with a fetal alcohol spectrum disorder in adulthood: Caregiver perspectives. *Journal on Developmental Disabilities, 24*(2), 29-42. http://ovidsp.ovid.com/ovidweb.cgi?T=JS&PAGE=reference&D=psyc17&NEWS=N&AN=2020-61329-004

Brown, J., Wiendels, S., Eyre, V., & Ali, A. (2019). Social justice competencies for counselling and psychotherapy: Perceptions of experienced practitioners and implications for contemporary practice. *Counselling & Psychotherapy Research, 19*(4), 533-543. https://doi.org/http://dx.doi.org/10.1002/capr.12247

Bruder, A. M., Crossley, K. M., Mosler, A. B., Patterson, B., Haberfield, M., & Donaldson, A. (2020). Co-creation of a sport-specific anterior cruciate ligament injury risk reduction program for women: A concept mapping approach. *Journal of science and medicine in sport, 23*(4), 353-360. https://doi.org/https://dx.doi.org/10.1016/j.jsams.2019.10.019

Busija, L., Cinelli, R., Toombs, M. R., Easton, C., Hampton, R., Holdsworth, K., Macleod, A., Nicholson, G. C., Nasir, B. F., Sanders, K. M., & McCabe, M. P. (2020). The Role of Elders in the Wellbeing of a Contemporary Australian Indigenous Community. *Gerontologist, 60*(3), 513-524. https://doi.org/10.1093/geront/gny140

Caarls, P. J., van Schijndel, M. A., Berk, G. v. d., Boenink, A. D., Boerman, D., Lijmer, J. G., Honig, A., Terra, M., Thijs, A., Verwey, B., Waarde, J. A. v., Wijngaarden, J. v., Busschbach, J. J. v., & Group, M. P. U. S. (2019). Factors influencing the admission

decision for Medical Psychiatry Units: A concept mapping approach. *PLoS ONE, 14*(9), e0221807. https://doi.org/https://dx.doi.org/10.1371/journal.pone.0221807

Cardwell, R., McKenna, L., Davis, J., & Gray, R. (2021). How is clinical credibility defined in nursing? A concept mapping study. *Journal of Clinical Nursing*, No-Specified. https://doi.org/http://dx.doi.org/10.1111/jocn.15572

Chakraborty, A., Howard, N. J., Daniel, M., Chong, A., Slavin, N., Brown, A., & Cargo, M. (2021). Prioritizing Built Environmental Factors to Tackle Chronic and Infectious Diseases in Remote Northern Territory (NT) Communities of Australia: A Concept Mapping Study. *International journal of environmental research and public health, 18*(10). https://doi.org/https://dx.doi.org/10.3390/ijerph18105178

Chan, C. W. H., Choi, K. C., Chan, H. Y. L., Wong, M. M. H., Ling, G. C. C., Chow, K. M., Chow, A. Y. M., Lo, R., Sham, M. M. K., & Alano, B. (2019). Unfolding and displaying the influencing factors of advance directives from the stakeholder's perspective: A concept mapping approach. *Journal of Advanced Nursing, 75*(7), 1549-1562. https://doi.org/http://dx.doi.org/10.1111/jan.14017

Chow, K. M., Chan, C. W. H., Choi, K. C., White, I. D., Siu, K. Y., & Sin, W. H. (2021). A practice model of sexuality nursing care: a concept mapping approach. *Supportive care in cancer : official journal of the Multinational Association of Supportive Care in Cancer, 29*(3), 1663-1673. https://doi.org/https://dx.doi.org/10.1007/s00520-020-05660-1

Cook, K. A., & Bergeron, K. (2020). Palliative care for young adults with life-limiting conditions: public health recommendations. *BMJ supportive & palliative care*. https://doi.org/https://dx.doi.org/10.1136/bmjspcare-2019-002042

D'Alonzo, K. T., Vilaro, F. M., Joseph, M. E., Oyeneye, V., Garsman, L., Rosas, S. R., Castaneda, M., & Vivar, M. (2020). Using Concept Mapping within a Community-Academic Partnership to Examine Obesity among Mexican Immigrants. *Progress in community health partnerships : research, education, and action, 14*(2), 173-185. https://doi.org/https://dx.doi.org/10.1353/cpr.2020.0016

de Boer, M. E., Depla, M. F. I. A., Frederiks, B. J. M., Negenman, A. A., Habraken, J. M., van Randeraad-van der Zee, C. H., Embregts, P. J. C. M., & Hertogh, C. M. P. M. (2019). Involuntary care-Capturing the experience of people with dementia in nursing homes. A concept mapping study. *Aging & Mental Health, 23*(4), 498-506. https://doi.org/http://dx.doi.org/10.1080/13607863.2018.1428934

Donaldson, A., Callaghan, A., Bizzini, M., Jowett, A., Keyzer, P., & Nicholson, M. (2019). A concept mapping approach to identifying the barriers to implementing an evidence-based sports injury prevention programme. *Injury prevention : journal of the*

International Society for Child and Adolescent Injury Prevention, 25(4), 244-251. https://doi.org/https://dx.doi.org/10.1136/injuryprev-2017-042639

Donaldson, A., Reimers, J. L., Brophy, K. T., & Nicholson, M. (2019). Barriers to rejecting junk food sponsorship in sport-a formative evaluation using concept mapping. *Public Health, 166*, 1-9. https://doi.org/https://dx.doi.org/10.1016/j.puhe.2018.09.021

Dopp, A. R., Parisi, K. E., Munson, S. A., & Lyon, A. R. (2020). Aligning implementation and user-centered design strategies to enhance the impact of health services: results from a concept mapping study. *Implementation science communications, 1*, 17. https://doi.org/https://dx.doi.org/10.1186/s43058-020-00020-w

Du Bois, S. N., Guy, A. A., Manser, K. A., Thomas, N. N., Noble, S., Lewis, R., Toles, J., Spivey, C., Khan, H., & Tully, T. (2020). Developing prepare2thrive, a community-based intervention targeting treatment engagement among african american individuals living with hiv and serious mental illness. *AIDS Care*, No-Specified. https://doi.org/http://dx.doi.org/10.1080/09540121.2020.1717420

Dulin, A. J., Earnshaw, V. A., Dale, S. K., Carey, M. P., Fava, J. L., Wilson-Barthes, M., Mugavero, M. J., Dougherty-Sheff, S., Johnson, B., Napravnik, S., & Howe, C. J. (2021). A Concept Mapping Study to Understand Multilevel Resilience Resources Among African American/Black Adults Living with HIV in the Southern United States. *AIDS and Behavior, 25*(3), 773-786. https://doi.org/https://dx.doi.org/10.1007/s10461-020-03042-6

Dunlop, S., Lewis, N., Richardson, R., Thomas, S., Devonald-Morris, M., Pontin, D., & Wallace, C. (2020). Using group concept mapping to explore the complexities of managing children's care. *Nurse researcher*. https://doi.org/https://dx.doi.org/10.7748/nr.2020.e1696

Ebrahimi-Madiseh, A., Eikelboom, R. H., Bennett, R. J., Upson, G. S., Friedland, P. L., Swanepoel, D. W., Psarros, C., Lai, W. K., & Atlas, M. D. (2020). What Influences Decision-Making for Cochlear Implantation in Adults? Exploring Barriers and Drivers From a Multistakeholder Perspective. *Ear and hearing, 41*(6), 1752-1763. https://doi.org/https://dx.doi.org/10.1097/AUD.0000000000000895

Eigeland, J. A., Jones, L., Sheeran, N., & Moffitt, R. L. (2021). Critical physician behaviors in the formation of a good physician-patient relationship: Concept mapping the perspective of patients with chronic conditions. *Patient Education and Counseling*, No-Specified. https://doi.org/http://dx.doi.org/10.1016/j.pec.2021.04.035

Esmaeili, N., & Bamdad Soofi, J. (2021). Expounding the knowledge conversion processes within the occupational safety and health management system (OSH-MS) using

concept mapping. *International journal of occupational safety and ergonomics : JOSE*, 1-16. https://doi.org/https://dx.doi.org/10.1080/10803548.2020.1853957

Ettinger, A. K., Ray, K. N., Burke, J. G., Thompson, J., Navratil, J., Chavis, V., Cole, S., Jenks, T., & Miller, E. (2021). A Community Partnered Approach for Defining Child and Youth Thriving. *Academic pediatrics, 21*(1), 53-62. https://doi.org/https://dx.doi.org/10.1016/j.acap.2020.04.011

Felx, A., Kane, M., Corbiere, M., & Lesage, A. (2020). Using Group Concept Mapping to Develop a Conceptual Model of Housing and Community-Based Residential Settings for Adults With Severe Mental Illness. *Frontiers in psychiatry, 11*, 430. https://doi.org/https://dx.doi.org/10.3389/fpsyt.2020.00430

Forsdike, K., Donaldson, A., & Seal, E. (2020). Responding to Violence Against Women in Sport: Challenges Facing Sport Organizations in Victoria, Australia. *Research quarterly for exercise and sport*, 1-16. https://doi.org/https://dx.doi.org/10.1080/02701367.2020.1844857

Gausman, J., Lloyd, D., Kallon, T., Subramanian, S. V., Langer, A., & Austin, S. B. (2019). Clustered risk: An ecological understanding of sexual activity among adolescent boys and girls in two urban slums in Monrovia, Liberia. *Social Science & Medicine, 224*, 106-115. https://doi.org/http://dx.doi.org/10.1016/j.socscimed.2019.02.010

Gausman, J., Othman, A., Daas, I., Hamad, I., Dabobe, M., & Langer, A. (2021). How Jordanian and Syrian youth conceptualise their sexual and reproductive health needs: a visual exploration using concept mapping. *Culture, Health & Sexuality, 23*(2), 176-191. https://doi.org/https://dx.doi.org/10.1080/13691058.2019.1698769

Glista, D., O'Hagan, R., Moodie, S., & Scollie, S. (2021). An examination of clinical uptake factors for remote hearing aid support: a concept mapping study with audiologists. *International Journal of Audiology, 60*(sup1), S13-S22. https://doi.org/https://dx.doi.org/10.1080/14992027.2020.1795281

Green, C., Walkup, J. T., Bostwick, S., & Trochim, W. (2019). Advancing the agenda in pediatric mental health education. *Pediatrics, 144*(3). https://doi.org/http://dx.doi.org/10.1542/peds.2018-2596

Guilcher, S. J. T., Cadel, L., Everall, A. C., Wiese, J. L., Hamilton-Wright, S., Salmon, C. C., & Matheson, F. I. (2020). Factors related to screening for problem gambling among healthcare and social service providers in Ontario, Canada: A concept mapping study. *Health & Social Care in the Community, 28*(3), 791-802. https://doi.org/https://dx.doi.org/10.1111/hsc.12909

Hart, K. M., & Neil, N. (2021). Down syndrome caregivers' support needs: a mixed-method participatory approach. *Journal of intellectual disability research : JIDR, 65*(1), 60-76. https://doi.org/https://dx.doi.org/10.1111/jir.12791

Hiler, M., Spindle, T. R., Dick, D., Eissenberg, T., Breland, A., & Soule, E. (2020). Reasons for transition from electronic cigarette use to cigarette smoking among young adult college students. *Journal of Adolescent Health, 66*(1), 56-63. https://doi.org/http://dx.doi.org/10.1016/j.jadohealth.2019.09.003

Hvidt, N. C., Nielsen, K. T., Korup, A. K., Prinds, C., Hansen, D. G., Viftrup, D. T., Assing Hvidt, E., Hammer, E. R., Falko, E., Locher, F., Boelsbjerg, H. B., Wallin, J. A., Thomsen, K. F., Schroder, K., Moestrup, L., Nissen, R. D., Stewart-Ferrer, S., Stripp, T. K., Steenfeldt, V. O., Sondergaard, J., & Waehrens, E. E. (2020). What is spiritual care? Professional perspectives on the concept of spiritual care identified through group concept mapping. *BMJ open, 10*(12), e042142. https://doi.org/https://dx.doi.org/10.1136/bmjopen-2020-042142

Jenkins, A. M., Burns, D., Horick, R., Spicer, B., Vaughn, L. M., & Woodward, J. (2021). Adolescents and Young Adults With Spina Bifida Transitioning to Adulthood: A Comprehensive Community-Based Needs Assessment. *Academic pediatrics*. https://doi.org/https://dx.doi.org/10.1016/j.acap.2021.02.002

Johnson, S., Van Hoye, A., Donaldson, A., Lemonnier, F., Rostan, F., & Vuillemin, A. (2020). Building health-promoting sports clubs: a participative concept mapping approach. *Public Health, 188*, 8-17. https://doi.org/https://dx.doi.org/10.1016/j.puhe.2020.08.029

Kabukye, J. K., de Keizer, N., & Cornet, R. (2020). Elicitation and prioritization of requirements for electronic health records for oncology in low resource settings: A concept mapping study. *International journal of medical informatics, 135*, 104055. https://doi.org/https://dx.doi.org/10.1016/j.ijmedinf.2019.104055

Kazmerski, T. M., Prushinskaya, O. V., Hill, K., Nelson, E., Leonard, J., Mogren, K., Pitts, S. A. B., Roboff, J., Uluer, A., Emans, S. J., Miller, E., & Sawicki, G. S. (2019). Sexual and Reproductive Health of Young Women With Cystic Fibrosis: A Concept Mapping Study. *Academic pediatrics, 19*(3), 307-314. https://doi.org/https://dx.doi.org/10.1016/j.acap.2018.08.011

Keller, A. O. (2021). A Healthy Life for African American Women Caregivers: A Concept Mapping Study. *Women's health issues : official publication of the Jacobs Institute of Women's Health, 31*(2), 130-139. https://doi.org/https://dx.doi.org/10.1016/j.whi.2020.11.011

Kiran, T., Wells, D., Okrainec, K., Kennedy, C., Devotta, K., Mabaya, G., Phillips, L., Lang, A., & O'Campo, P. (2020). Patient and caregiver priorities in the transition from hospital to home: results from province-wide group concept mapping. *BMJ quality & safety, 29*(5), 390-400. https://doi.org/https://dx.doi.org/10.1136/bmjqs-2019-009993

Leyns, C. C., Couvreur, N., Willems, S., & Van Hecke, A. (2021). Needs and resources of people with type 2 diabetes in peri-urban Cochabamba, Bolivia: a people-centred perspective. *International journal for equity in health, 20*(1), 104. https://doi.org/https://dx.doi.org/10.1186/s12939-021-01442-1

Macleod, A., Busija, L., & McCabe, M. (2020). Mapping the Perceived Sexuality of Heterosexual Men and Women in Mid- and Later Life: A Mixed-Methods Study. *Sexual medicine, 8*(1), 84-99. https://doi.org/https://dx.doi.org/10.1016/j.esxm.2019.10.001

Mahabir, D. F., O'Campo, P., Lofters, A., Shankardass, K., Salmon, C., & Muntaner, C. (2021). Classism and Everyday Racism as Experienced by Racialized Health Care Users: A Concept Mapping Study. *International journal of health services : planning, administration, evaluation*, 207314211014782. https://doi.org/https://dx.doi.org/10.1177/00207314211014782

Mahabir, D. F., O'Campo, P., Lofters, A., Shankardass, K., Salmon, C., & Muntaner, C. (2021). Experiences of everyday racism in Toronto's health care system: a concept mapping study. *International journal for equity in health, 20*(1), 74. https://doi.org/https://dx.doi.org/10.1186/s12939-021-01410-9

McCaffrey, S. A., Chiauzzi, E., Chan, C., & Hoole, M. (2019). Understanding 'good health care' from the patient's perspective: Development of a conceptual model using group concept mapping. *The Patient: Patient-Centered Outcomes Research, 12*(1), 83-95. https://doi.org/http://dx.doi.org/10.1007/s40271-018-0320-x

McMahon, S., Burnham, J., & Banyard, V. L. (2020). Bystander intervention as a prevention strategy for campus sexual violence: Perceptions of historically minoritized college students. *Prevention Science*, No-Specified. https://doi.org/http://dx.doi.org/10.1007/s11121-020-01134-2

McNeish, R., Walker, C., Massey, O., & Tran, Q. (2020). Using concept mapping to operationalize mental well-being for men and boys. *American Journal of Community Psychology, 66*(1-2), 14-23. https://doi.org/http://dx.doi.org/10.1002/ajcp.12419

Melvin, K., Meyer, C., & Scarinci, N. (2020). What does a family who is "engaged" in early intervention look like? Perspectives of Australian speech-language pathologists.

International Journal of Speech-Language Pathology, 1-11. https://doi.org/https://dx.doi.org/10.1080/17549507.2020.1784279

Nielsen, K. T., Klokker, L., Guidetti, S., & Waehrens, E. E. (2019). Identifying, organizing and prioritizing ideas on how to enhance ADL ability. *Scandinavian journal of occupational therapy, 26*(5), 382-393. https://doi.org/https://dx.doi.org/10.1080/11038128.2018.1424235

Nielsen, K. T., Rasmussen, M. U., Overgaard, A. F., Klokker, L., Christensen, R., & Waehrens, E. E. (2020). Identifying values and preferences around the choice of analgesia for patients with acute trauma pain in emergency and prehospital settings: using group concept mapping methodology. *BMJ open, 10*(3), e031863. https://doi.org/https://dx.doi.org/10.1136/bmjopen-2019-031863

Nijs, S., Taminiau, E. F., Frielink, N., & Embregts, P. J. C. M. (2019). Stakeholders' perspectives on how to improve the support for persons with an intellectual disability and challenging behaviors: A concept mapping study. *International Journal of Developmental Disabilities*, No-Specified. https://doi.org/http://dx.doi.org/10.1080/20473869.2019.1690859

Noel Racine, A., Garbarino, J. M., Corrion, K., D'Arripe-Longueville, F., Massiera, B., & Vuillemin, A. (2020). Perceptions of barriers and levers of health-enhancing physical activity policies in mid-size French municipalities. *Health research policy and systems, 18*(1), 62. https://doi.org/https://dx.doi.org/10.1186/s12961-020-00575-z

Nuampa, S., Tilokskulchai, F., Patil, C. L., Sinsuksai, N., & Phahuwatanakorn, W. (2019). Factors related to exclusive breastfeeding in Thai adolescent mothers: Concept mapping approach. *Maternal & child nutrition, 15*(2), e12714. https://doi.org/https://dx.doi.org/10.1111/mcn.12714

Pearson, T., Chandler, R., McCreary, L. L., Patil, C. L., & McFarlin, B. L. (2020). Perceptions of African American Women and Health Care Professionals Related to Pre-Exposure Prophylaxis to Prevent HIV. *Journal of obstetric, gynecologic, and neonatal nursing : JOGNN, 49*(6), 571-580. https://doi.org/https://dx.doi.org/10.1016/j.jogn.2020.07.003

Phad, A., Johnston, S., Tabak, R. G., Mazzucca, S., & Haire-Joshu, D. (2019). Developing priorities to achieve health equity through diabetes translation research: a concept mapping study. *BMJ open diabetes research & care, 7*(1), e000851. https://doi.org/https://dx.doi.org/10.1136/bmjdrc-2019-000851

Piedra, L. M., Ridings, J., Howe, M. J. K., Smith, J. L., O'Brien, C., Howard, A., & Conrad, K. J. (2020). Stakeholders' Ideas About Positive Aging for Latinos: A Conceptual Map.

Journal of applied gerontology : the official journal of the Southern Gerontological Society, 733464820935749. https://doi.org/https://dx.doi.org/10.1177/0733464820935749

Rachlis, B., Nam, S., Rosenes, R., Santoni, T., Peck, R., Betts, A., Kendall, C., Yoong, D., Sharp, A., Gauvin, H., Goddard, L., Owino, M., Rourke, S. B., & Antoniou, T. (2021). Using concept mapping to explore the challenges associated with affording and accessing medications among people living with HIV in Ontario, Canada. *AIDS Care, 33*(6), 827-832. https://doi.org/10.1080/09540121.2020.1770182

Rising, K. L., Doyle, S. K., Powell, R. E., Doty, A. M. B., LaNoue, M., & Gerolamo, A. M. (2019). Use of Group Concept Mapping to Identify Patient Domains of Uncertainty That Contribute to Emergency Department Use. *Journal of emergency nursing, 45*(1), 46-53. https://doi.org/https://dx.doi.org/10.1016/j.jen.2018.05.015

Robinson, L. J., Stephens, N. M., Wilson, S., Graham, L., & Hackett, K. L. (2020). Conceptualizing the key components of rehabilitation following major musculoskeletal trauma: A mixed methods service evaluation. *Journal of evaluation in clinical practice, 26*(5), 1436-1447. https://doi.org/https://dx.doi.org/10.1111/jep.13331

Roodenrijs, N. M. T., van der Goes, M. C., Welsing, P. M. J., van Oorschot, E. P. C., Nikiphorou, E., Nijhof, N. C., Tekstra, J., Lafeber, F. P. J. G., Jacobs, J. W. G., van Laar, J. M., & Geenen, R. (2021). Non-adherence in difficult-to-treat rheumatoid arthritis from the perspectives of patients and rheumatologists: a concept mapping study. *Rheumatology (Oxford, England)*. https://doi.org/https://dx.doi.org/10.1093/rheumatology/keab130

Rostad-Tollefsen, H. K., Kolset, S. O., Retterstol, K., Hesselberg, H., & Nordstrom, M. (2021). Factors influencing the opportunities of supporting staff to promote a healthy diet in adults with intellectual disabilities. *Journal of applied research in intellectual disabilities : JARID, 34*(3), 733-741. https://doi.org/https://dx.doi.org/10.1111/jar.12846

Rouhi, M., Stirling, C. M., & Crisp, E. P. (2019). Mothers' views of health problems in the 12 months after childbirth: A concept mapping study. *Journal of Advanced Nursing, 75*(12), 3702-3714. https://doi.org/https://dx.doi.org/10.1111/jan.14187

Singer, B. A., Keith, S., Howerter, A., Doll, H., Pham, T., & Mehta, R. (2021). A Study Comparing Patient and Clinician Perspectives of Treatments for Multiple Sclerosis via Group Concept Mapping. *Patient preference and adherence, 15*, 975-987. https://doi.org/https://dx.doi.org/10.2147/PPA.S297052

Smith, F., Alexandersson, P., Bergman, B., Vaughn, L., & Hellstrom, A. (2019). Fourteen years of quality improvement education in healthcare: a utilisation-focused evaluation using concept mapping. *BMJ open quality, 8*(4), e000795. https://doi.org/https://dx.doi.org/10.1136/bmjoq-2019-000795

Smith, F., Gunnarsdottir, K. A., Genell, A., McLinden, D., Vaughn, L., Garelius, H., Nilsson-Ehle, H., Lonqvist, U., & Bjork-Eriksson, T. (2019). Evaluating the implementation and use of the regional cancer plan in Western Sweden through concept mapping. *International journal for quality in health care : journal of the International Society for Quality in Health Care, 31*(7), 44-52. https://doi.org/https://dx.doi.org/10.1093/intqhc/mzy241

Sommerfeld, D. H., Jaramillo, E. T., Lujan, E., Haozous, E., & Willging, C. E. (2021). Health Care Access and Utilization for American Indian Elders: A Concept-Mapping Study. *The journals of gerontology. Series B, Psychological sciences and social sciences, 76*(1), 141-151. https://doi.org/https://dx.doi.org/10.1093/geronb/gbz112

Soule, E. K., Lee, J. G. L., Egan, K. L., Bode, K. M., Desrosiers, A. C., Guy, M. C., Breland, A., & Fagan, P. (2020). "I cannot live without my vape": Electronic cigarette user-identified indicators of vaping dependence. *Drug and Alcohol Dependence, 209*. https://doi.org/http://dx.doi.org/10.1016/j.drugalcdep.2020.107886

Soule, E. K., Mayne, S., Snipes, W., Guy, M. C., Breland, A., & Fagan, P. (2020). Impacts of COVID-19 on Electronic Cigarette Purchasing, Use and Related Behaviors. *International journal of environmental research and public health, 17*(18). https://doi.org/https://dx.doi.org/10.3390/ijerph17186762

Staley, K., Donaldson, A., Randle, E., Nicholson, M., O'Halloran, P., Nelson, R., & Cameron, M. (2019). Challenges for sport organisations developing and delivering non-traditional social sport products for insufficiently active populations. *Australian and New Zealand journal of public health, 43*(4), 373-381. https://doi.org/https://dx.doi.org/10.1111/1753-6405.12912

Strassheim, V., Deary, V., Webster, D. A., Douglas, J., Newton, J. L., & Hackett, K. L. (2021). Conceptualizing the benefits of a group exercise program developed for those with chronic fatigue: a mixed methods clinical evaluation. *Disability and rehabilitation, 43*(5), 657-667. https://doi.org/https://dx.doi.org/10.1080/09638288.2019.1636315

Svobodova, I., Filakovska Bobakova, D., Bosakova, L., & Dankulincova Veselska, Z. (2021). How to improve access to health care for Roma living in social exclusion: a concept mapping study. *International journal for equity in health, 20*(1), 61. https://doi.org/https://dx.doi.org/10.1186/s12939-021-01396-4

Sweegers, M. G., Buffart, L. M., van Veldhuizen, W. M., Geleijn, E., Verheul, H. M. W., Brug, J., Chinapaw, M. J. M., & Altenburg, T. M. (2019). How Does a Supervised Exercise Program Improve Quality of Life in Patients with Cancer? A Concept Mapping Study Examining Patients' Perspectives. *The oncologist, 24*(6), e374-e383. https://doi.org/https://dx.doi.org/10.1634/theoncologist.2017-0613

Thepha, T., Marais, D., Bell, J., & Muangpin, S. (2019). Concept mapping to reach consensus on a 6-month exclusive breastfeeding strategy model to improve the rate in Northeast Thailand. *Maternal & child nutrition, 15*(4), e12823. https://doi.org/https://dx.doi.org/10.1111/mcn.12823

Urbanoski, K., Pauly, B., Inglis, D., Cameron, F., Haddad, T., Phillips, J., Phillips, P., Rosen, C., Schlotter, G., Hartney, E., & Wallace, B. (2020). Defining culturally safe primary care for people who use substances: a participatory concept mapping study. *BMC health services research, 20*(1), 1060. https://doi.org/https://dx.doi.org/10.1186/s12913-020-05915-x

Vaughn, L. M., Sunny, C. E., Lindquist-Grantz, R., King, C., Brent, D., Boyd, S., & Grupp-Phelan, J. (2020). Successful Suicide Screening in the Pediatric Emergency Department: Youth, Parent, Researcher, and Clinician Perspectives. *Archives of suicide research : official journal of the International Academy for Suicide Research, 24*(sup1), 124-141. https://doi.org/https://dx.doi.org/10.1080/13811118.2018.1541034

Velonis, A. J., Hebert-Beirne, J., Conroy, L. M., Hernandez, M., Castaneda, D., & Forst, L. (2020). Impact of precarious work on neighborhood health: Concept mapping by a community/academic partnership. *American journal of industrial medicine, 63*(1), 23-35. https://doi.org/https://dx.doi.org/10.1002/ajim.23055

Visek, A. J., Blake, E. F., Otterbein, M., Chandran, A., & Sylvetsky, A. C. (2019). SWEET MAPS: A Conceptualization of Low-Calorie Sweetener Consumption Among Young Adults. *Current developments in nutrition, 3*(4), nzy103. https://doi.org/https://dx.doi.org/10.1093/cdn/nzy103

Walker, D. C., Heiss, S., Donahue, J. M., & Brooks, J. M. (2020). Practitioners' perspectives on ethical issues within the treatment of eating disorders: Results from a concept mapping study. *International Journal of Eating Disorders, 53*(12), 1941-1951. https://doi.org/http://dx.doi.org/10.1002/eat.23381

Washington-Nortey, P.-M., & Serpell, Z. (2021). Parental expectations for children with intellectual disability or autism in Ghana and Zambia: A concept mapping study. *Research in Developmental Disabilities, 114*, 103989. https://doi.org/https://dx.doi.org/10.1016/j.ridd.2021.103989

Wentink, C., Huijbers, M. J., Lucassen, P. L., van der Gouw, A., Kramers, C., Spijker, J., & Speckens, A. E. (2019). Enhancing shared decision making about discontinuation of antidepressant medication: a concept-mapping study in primary and secondary mental health care. *The British journal of general practice : the journal of the Royal College of General Practitioners, 69*(688), e777-e785. https://doi.org/https://dx.doi.org/10.3399/bjgp19X706001
