# Supplementary material for: A Systematic Review to Inform the Development of a Reporting Guideline for Concept Mapping Research
Source: Methods Protoc. 2023 Oct 17;6(5):101. doi: 10.3390/mps6050101 (PMC10609252; doi:10.3390/mps6050101)
Supplement: Supplementary file 1 [file mps-06-00101-s001.zip › Supplementary document 7_List of 13 excluded studies.pdf]

List of studies excluded during data extraction

Donaldson, A., Reimers, J. L., Brophy, K. T., & Nicholson, M. (2019). Barriers to rejecting junk food sponsorship in sport-a formative evaluation using concept mapping. *Public Health, 166*, 1-9. https://doi.org/https://dx.doi.org/10.1016/j.puhe.2018.09.021

Dopp, A. R., Parisi, K. E., Munson, S. A., & Lyon, A. R. (2020). Aligning implementation and user-centered design strategies to enhance the impact of health services: results from a concept mapping study. *Implementation science communications, 1*, 17. https://doi.org/https://dx.doi.org/10.1186/s43058-020-00020-w

Felx, A., Kane, M., Corbiere, M., & Lesage, A. (2020). Using Group Concept Mapping to Develop a Conceptual Model of Housing and Community-Based Residential Settings for Adults With Severe Mental Illness. *Frontiers in psychiatry, 11*, 430. https://doi.org/https://dx.doi.org/10.3389/fpsyt.2020.00430

Forsdike, K., Donaldson, A., & Seal, E. (2020). Responding to Violence Against Women in Sport: Challenges Facing Sport Organizations in Victoria, Australia. *Research quarterly for exercise and sport*, 1-16. https://doi.org/https://dx.doi.org/10.1080/02701367.2020.1844857

Hiler, M., Spindle, T. R., Dick, D., Eissenberg, T., Breland, A., & Soule, E. (2020). Reasons for transition from electronic cigarette use to cigarette smoking among young adult college students. *Journal of Adolescent Health, 66*(1), 56-63. https://doi.org/http://dx.doi.org/10.1016/j.jadohealth.2019.09.003

Hvidt, N. C., Nielsen, K. T., Korup, A. K., Prinds, C., Hansen, D. G., Viftrup, D. T., Assing Hvidt, E., Hammer, E. R., Falko, E., Locher, F., Boelsbjerg, H. B., Wallin, J. A., Thomsen, K. F., Schroder, K., Moestrup, L., Nissen, R. D., Stewart-Ferrer, S., Stripp, T. K., Steenfeldt, V. O., Sondergaard, J., & Waehrens, E. E. (2020). What is spiritual care? Professional perspectives on the concept of spiritual care identified through group concept mapping. *BMJ open, 10*(12), e042142. https://doi.org/https://dx.doi.org/10.1136/bmjopen-2020-042142

Kabukye, J. K., de Keizer, N., & Cornet, R. (2020). Elicitation and prioritization of requirements for electronic health records for oncology in low resource settings: A concept mapping study. *International journal of medical informatics, 135*, 104055. https://doi.org/https://dx.doi.org/10.1016/j.ijmedinf.2019.104055

Mahabir, D. F., O'Campo, P., Lofters, A., Shankardass, K., Salmon, C., & Muntaner, C. (2021). Classism and Everyday Racism as Experienced by Racialized Health Care Users: A Concept Mapping Study. *International journal of health services : planning,*

administration, evaluation, 207314211014782. https://doi.org/https://dx.doi.org/10.1177/00207314211014782

McMahon, S., Burnham, J., & Banyard, V. L. (2020). Bystander intervention as a prevention strategy for campus sexual violence: Perceptions of historically minoritized college students. *Prevention Science*, No-Specified. https://doi.org/http://dx.doi.org/10.1007/s11121-020-01134-2

Noel Racine, A., Garbarino, J. M., Corrion, K., D'Arripe-Longueville, F., Massiera, B., & Vuillemin, A. (2020). Perceptions of barriers and levers of health-enhancing physical activity policies in mid-size French municipalities. *Health research policy and systems, 18*(1), 62. https://doi.org/https://dx.doi.org/10.1186/s12961-020-00575-z

Piedra, L. M., Ridings, J., Howe, M. J. K., Smith, J. L., O'Brien, C., Howard, A., & Conrad, K. J. (2020). Stakeholders' Ideas About Positive Aging for Latinos: A Conceptual Map. *Journal of applied gerontology : the official journal of the Southern Gerontological Society*, 733464820935749. https://doi.org/https://dx.doi.org/10.1177/0733464820935749

Smith, F., Alexandersson, P., Bergman, B., Vaughn, L., & Hellstrom, A. (2019). Fourteen years of quality improvement education in healthcare: a utilisation-focused evaluation using concept mapping. *BMJ open quality, 8*(4), e000795. https://doi.org/https://dx.doi.org/10.1136/bmjoq-2019-000795

Staley, K., Donaldson, A., Randle, E., Nicholson, M., O'Halloran, P., Nelson, R., & Cameron, M. (2019). Challenges for sport organisations developing and delivering non-traditional social sport products for insufficiently active populations. *Australian and New Zealand journal of public health, 43*(4), 373-381. https://doi.org/https://dx.doi.org/10.1111/1753-6405.12912
